# Human Alveolar and Splenic Macrophage Populations Display a Distinct Transcriptomic Response to Infection With *Mycobacterium tuberculosis*

**DOI:** 10.3389/fimmu.2020.00630

**Published:** 2020-04-21

**Authors:** Lelia Lavalett, Hector Ortega, Luis F. Barrera

**Affiliations:** ^1^Grupo de Inmunología Celular e Inmunogenética, Facultad de Medicina, Instituto de Investigaciones Médicas, Universidad de Antioquia, Medellín, Colombia; ^2^Facultad de Ciencias, Universidad Nacional de Colombia Sede Medellín, Medellín, Colombia; ^3^Clínica Cardiovascular Santa María, Medellín, Colombia; ^4^Facultad de Medicina, Universidad de Antioquia, Medellín, Colombia

**Keywords:** human alveolar macrophages, splenic macrophages, *M. tuberculosis*, clinical isolates, microarray, transcriptome

## Abstract

*Mycobacterium tuberculosis* (Mtb) infects alveolar macrophages (AMs), causing pulmonary tuberculosis (PTB), the most common form of the disease. Less frequently, Mtb is disseminated to many other organs and tissues, resulting in different extrapulmonary forms of TB. Nevertheless, very few studies have addressed the global mRNA response of human AMs, particularly from humans with the active form of the disease. Strikingly, almost no studies have addressed the response of human extrapulmonary macrophages to Mtb infection. In this pilot study, using microarray technology, we examined the transcriptomic *ex vivo* response of AMs from PTB patients (AMTBs) and AMs from control subjects (AMCTs) infected with two clinical isolates of Mtb. Furthermore, we also studied the infection response of human splenic macrophages (SMs) to Mtb isolates, as a model for extrapulmonary infection, and compared the transcriptomic response between AMs and SMs. Our results showed a striking difference in global mRNA profiles in response to infection between AMs and SMs, implicating a tissue-specific macrophage response to Mtb.

## Introduction

Different cell populations of the mononuclear phagocytic system (MPS), including macrophages and monocytes, are preferred targets of *Mycobacterium tuberculosis* (Mtb), the agent causing human tuberculosis (TB). TB is considered one of the deadliest infectious diseases in human history (1). For the year 2017, cases of TB reached 10.4 million worldwide, from which 6.7 million were considered new cases, and 16% were extrapulmonary TB cases, amounting to 1.6 million deaths of HIV-negative and -positive people, overall. Of interest is the observation that 1.7 billion people are suspected to be latently infected with Mtb and are at higher risk of developing the active form of the disease (2).

*Mycobacterium tuberculosis* infection is transmitted by an aerosol route from patients with active TB to their contacts, mainly household contacts. The bacillus is then transported to the lung alveoli, where alveolar macrophages (AMs) are considered the main targets of the initial infection (3). However, in some circumstances, Mtb can be disseminated to other organs and tissues, causing different extrapulmonary TB forms of the disease (4).

Based on the mouse model of Mtb aerosol infection, a complex series of events occurs in infected macrophages (5). AMs recognize different pathogen-associated molecular patterns (PAMPs) present or secreted by Mtb using pattern recognition receptors (PRRs), leading to an initial innate immune response to control the infection (6–8).

Infected AMs cross the interstitial space of the lung, disseminating the infection to interstitial macrophages and other migratory myeloid populations, including monocytes (9, 10). The recruitment of additional cell types gives rise to granuloma formation, a structure associated with protection against dissemination (11). In a minority of infected individuals, the granuloma is unable to contain the infection; Mtb heavily replicates and may disseminate to other organs and tissues, leading to extrapulmonary infections and extrapulmonary TB (12). Interestingly, DNA from Mtb has been recovered from the spleen and kidney of healthy deceased individuals from causes other than TB (13), suggesting that Mtb dissemination may take place in people with latent TB infection.

Although the results of previous studies have provided a framework for understanding the consequences of macrophage–Mtb interaction, relatively little data are available regarding human AMs collected from healthy individuals (14, 15), while no data have been obtained from AMs from TB patients. So far, most of our knowledge from the interaction of Mtb with MPS cells has been garnered by the use of monocyte-derived macrophages (MDMs) from healthy individuals (14, 16–18).

Gene expression profiles from TB patients and uninfected healthy controls have been reported by several groups in the past to provide new knowledge on the immune response that takes place during active TB (19). In comparison, tissue samples from extrapulmonary TB have been poorly studied (20, 21). Genome-wide expression profiling studies are essential to provide critical clues to understand the complexities of the immune response to mycobacterial infections, to identify important genes and pathways in infected cells, and to generate new biomarkers of disease prognosis and diagnosis (22).

In this report, we present evidence of a genome-wide microarray expression profiling of AMs from healthy individuals and TB patients, as well as splenic macrophages (SMs) from deceased individuals from causes other than TB and those infected *in vitro* with two Mtb clinical isolates (UT127 and UT205), whose genomes were recently sequenced to show differential determinants of virulence (23, 24). Our results showed that AMTBs display an attenuated transcriptomic response to Mtb infection, compared to AMCTs without active TB. AMTBs regulate genes associated with interferon (IFN)-signaling pathways, and several critical pathways in active TB were also induced, such as the inflammasome (*AIM2*), FC pathway receptor (*FCGR1A*), and myeloid inflammatory pathway (*TREM1*). These genes and signaling pathways could be novel and of interest to understand certain critical aspects of the pathophysiology of TB. Surprisingly, the analysis of the mRNA profiles of the SMs infected with UT127 or UT205 exhibited a lower transcriptomic response compared to AMs, suggesting a clear-cut difference in the transcriptomic response between AMs and SMs in terms of their capacity to respond to Mtb.

## Materials and Methods

### Subjects

AMs from bacteriologically confirmed pulmonary TB patients (*n* = 4), two females (mean age: 26; range: 22–30), two males (mean age: 36; range: 25–47), and healthy controls (*n* = 4), one female (50 years old), and three males (mean age: 44; range: 19–46) were obtained from bronchoalveolar lavages (BALs) as previously described (25, 26). Non-smoking individuals as well as those testing negative for HIV, cancer, and diabetes at the time of sampling voluntarily participated in this study. BAL from TB patients was obtained within the first 2 weeks of anti-TB treatment. Human spleen slices from deceased donors of the Transplantation Program of the Hospital Universitario Pablo Tobón Uribe, and the IPS Universitaria León XIII Sede Medellín (Medellín, Colombia), specifically two females (mean age: 24.5; range: 23–26) and three males (mean age: 33; range: 28–35), were used to obtain the SMs. Trauma, including different cerebrovascular causes, was the cause of donor death. None of the donors tested positive for HIV.

### Mycobacteria

Mtb clinical isolates UT205 and UT127 were obtained from the Centro Colombiano para la Investigación en Tuberculosis (CCITB) from a cohort study of TB patients and their household contacts (27). The UT127 and UT205 strains were initially selected based on epidemiological evidence. While there was an incident case in the house of a TB patient infected with UT205, there were no such cases in the household of the TB patient with UT127. In addition, infection of human macrophages with UT127 mostly triggered apoptotic-like cell death, while UT205 mostly triggered necrotic-type cell death (25, 26). Mtb was cultured as previously described (28). Briefly, mycobacteria were grown in Middlebrook 7H9 broth (Difco, Detroit, USA, #295939) +emented with 10% oleic acid, albumin, dextrose, and catalase (OADC, Becton Dickinson, Franklin Lakes, USA, #211886) and 0.05% Tween 80 (Sigma, St. Louis, MO, USA, #59924) for 2–3 weeks to reach the exponential growth phase, and the clumps were disrupted by sonication (CV33 Sonics Vibra Cell, Newtown, USA). The bacterial suspension was diluted in freezing medium and frozen at −70°C until use. To determine the number of colony-forming units (CFU), 20 μl of each serial dilution was plated onto Petri dishes (Corning, NY, USA) containing Middlebrook 7H10 agar (Becton Dickinson, Franklin Lakes, NJ, USA, #254520) supplemented with glycerol and 10% OADC (Becton Dickinson, Franklin Lakes, NJ, USA, #211886) pH 7.2 and grown at 37°C for 3 weeks. Upon thawing, mycobacterial viability (usually more than 90%) was tested using fluorescein diacetate (FDA)-stained bacteria by flow cytometry (29). Both isolates belonged to the Latino American and Mediterranean family (LAM9) of Mtb (24, 30).

### Isolation of Macrophages

AMs were prepared as previously described (25, 26). In summary, fiber optic bronchoscopy was performed to obtain AMs from non-compromised lung areas of pulmonary tuberculosis (PTB). Single-cell suspensions were obtained after BAL and passed through a 40-μm cell strainer (Thermo Fisher, NH, #352340), centrifuged for 5 min at 4°C, after which 650 × *g* was harvested and resuspended in RPMI-1640 (Sigma, St. Louis, MO, USA, #R8758), supplemented with 10% AB+-inactivated human serum (Sigma, St. Louis, MO, USA, H5667) and antibiotics (complete medium). Viability (≥95%) was estimated by trypan blue exclusion and expressed as a percentage of the total cells recovered. Three hundred thousand dark granular cells, morphologically corresponding to macrophages, were seeded on 24-well plates and cultured for 4 days in complete medium at 37°C, 5% CO_2_, and 95% relative humidity. At this point, non-adherent cells were eliminated by extensive washings with pre-warmed (37°C) DPBS (Invitrogen, Grand Island, USA) supplemented with 0.5% AB+ inactivated human serum, and then cultured for an additional 24 h in complete medium without antibiotics before being infected.

SMs were obtained as previously described (31). Briefly, splenic mononuclear cell suspensions were obtained after mechanical disruption of spleen slices, lysis of red blood cells, and separation by density centrifugation on Ficoll Hypaque (Lymphoprep, Alere Technologies AS, Oslo, Norway, # 1114545), 400 × *g*, 30 min, 20°C. 400 × 10^6^ viable (higher than 90%) splenic mononuclear cells were cultured in 100 × 17 mm Dish, Nunclon™ Delta dishes (Thermo Fisher Scientific, Waltham, MA, USA, # 150350) in complete medium for 4 days. Adherent cells were detached by treatment with 0.05% trypsin–EDTA (Sigma, St. Louis, MO, USA, T3924), washed, counted, and then seeded at 3 × 10^5^ macrophages per well on 24-well tissue culture plates in complete medium. SMs were cultured in complete medium without antibiotics for 24 h before infection.

### Infection of Macrophages With Mtb

Macrophages were infected for 6 h with Mtb at a multiplicity of infection (MOI) of 10:1. The percentage of infected macrophages in the experimental conditions used in this study was defined in preliminary experiments using Kinyoun staining. At least 400 cells in randomly selected fields were counted. In all cases, the proportion of infected macrophages was >85% (data not shown). The cells were extensively washed with pre-warmed (37°C) DPBS supplemented with 0.5% AB+-inactivated human serum to eliminate non-ingested bacteria. Adherent cells were then lysed in RLT buffer (Qiagen, Carlsbad, USA) with 1% β-mercaptoethanol and stored at −80°C until use for RNA extraction. To account for changes in RNA expression profiles in each group of macrophages, three experimental settings were evaluated: (1) *in vitro* non-infected cells (NI) used as a control; (2) cells infected *in vitro* with Mtb UT127; and (3) cells infected *in vitro* with Mtb UT205.

### Isolation of RNA

Experimental variation in the number of cells seeded, adhered, or infected was controlled by extracting total RNA from three to five replica wells for each sample. A total of 39 RNA samples were obtained according to the experimental settings described earlier. However, some RNA samples were excluded from the analysis because they did not meet the quality requirements (see *Results* section). Total RNA was extracted as previously described (25, 26). Briefly, samples were treated with RLT buffer and kept at −80°C until further processing. RNA from all samples was prepared simultaneously to reduce sample-to-sample variability. RNA was extracted using the RNeasy Plus Mini Kit (Qiagen, Carlsbad, USA, #74134), which includes gDNA eliminator columns, following the manufacturer's protocol. RNA quality was assessed using a Nanodrop 2000 Spectrometer (Thermo Scientific, Waltham, USA) by measuring the ratio of absorbance at 260 and 280 nm, and the integrity of the RNA (RIN) was evaluated employing the Agilent 2100 Bioanalyzer (Agilent Technologies, Waldbronn, Germany). Only samples with a RIN >7 (average median RIN = 9.2; IQ = 0.8; range = 7.5–9.9) were selected for microarray analysis.

### Microarray Analyses

RNA expression analysis was performed using the Illumina Human BeadChip (Illumina, San Diego, USA), which provides coverage of 47,231 probes targeting more than 31,000 annotated genes, from which 22,283 are unique. Sample processing was performed at Macrogen (Seoul, Korea) as previously described (25, 26). In summary, total RNA was amplified and purified using the Ambion Illumina RNA amplification kit (Ambion, Austin, USA) to yield biotinylated cRNA according to the manufacturer's instructions. RNA (550 ng) was reverse-transcribed to cDNA using a T7 oligo (dT) primer. Second-strand cDNA was synthesized, transcribed *in vitro*, and labeled with biotin-NTP. According to the manufacturer's protocol, purified labeled cRNA (750 ng) was hybridized to each Human HT-12 v.4.0 bead array for 16–18 h at 58°C. The Amersham Fluorolink Streptavidin-Cy3 System (GE Healthcare Bio-Sciences, Little Chalfont, UK) was utilized to detect array signals following the bead array manual. A bead array confocal scanner was used to scan the arrays according to the manufacturer's instructions. Hybridization quality and overall chip performance were monitored by visual inspection of both internal quality control checks and the raw scanned data. Raw data were extracted with the Illumina GenomeStudio v2011.1 (Gene Expression Module v1.9.0). Probe signal values were transformed using the logarithm method. All data were submitted to the NCBI Gene Expression Omnibus (GEO) database, accession number GSE139825.

### Data Analysis

Data were analyzed as previously described (25, 26). Lumi Bioconductor package (32) was used to preprocess the raw data. This package includes a quality control initial check, a variance-stabilizing transformation (VST) step (33), in which the algorithm takes advantage of the technical replicates available on every Illumina microarray, and a normalization (quantile) step. Before the analysis of differentially expressed genes (DEGs), a filter was applied to minimize false positives and remove the unexpressed and unannotated genes. Absent/present call detection was performed using a *p* value of 0.01 as a threshold. Genes or probes with an expression level below the threshold value were removed across all groups.

Statistical analysis was performed with ~15,000 probes for each group that reached a significant value for detection. The Limma package of the Bioconductor environment (34) was used to identify DEGs in infected macrophages and they were compared to their respective uninfected controls. Stringent criteria, including Log_2_ of fold-change (Log_2_FC) ≥1.5 or ≤ 1.5, and false discovery rate (FDR < 0.05) were applied to filter the DEGs.

### Functional Enrichment and Canonical Pathway Analyses

Functional enrichment analysis was conducted to interpret the gene set of interest using Gene Ontology (GO) and the Kyoto Encyclopedia of Genes and Genomes (KEGG) pathways (35). The STRING database (36) was used to perform a functional association analysis using FDR <0.01. Ingenuity Pathway Analysis (IPA) (37) was used to classify DEGs according to their functional relationships, and show canonical pathways and networks involving these genes with potentially critical host mediators of TB disease. IPA includes canonical pathways described in the Ingenuity Pathways Knowledge Base using a built-in scientific literature-based database. The nodes within networks represent genes and lines indicate biological relationships (direct or indirect) with other genes rooted in published literature within the IPA software. The network score is based on the hypergeometric distribution and calculated using the right-tailed Fisher's exact test. Network-wise, IPA computes a score for each network related to the fit of the input set of DEGs. The *Z* score is obtained from the likelihood that the focus genes in a network are together by chance, with a *Z* score of 2 being equivalent to a *p* value of 0.01 (37).

### Technical Validation of Differential Expression Using Quantitative Real-Time PCR

Validation and confirmation of the microarray results were performed using quantitative real-time PCR (qRT-PCR). The *IL1B, TNFA, TNFAIP6*, and *IL8* genes were upregulated in AMCTs, AMTBs, and SMs in response to infection with both clinical isolates UT127 and UT205 in the microarray analysis and are important genes in the response of human macrophages to infection with Mtb (38). In this particular case, samples from three individuals from each group of macrophages (AMTBs, AMCTs, and SMs) were evaluated, including RNAs in duplicate of the three experimental settings for each group (NI, samples infected with Mtb UT127, and samples infected with Mtb UT205), which were previously analyzed in microarray experiments. Total RNA (100 ng) was reverse transcribed using SuperScript® III Platinum® Two-Step qRT-PCR Kit (Invitrogen, Carlsbad, USA), according to the manufacturer's instructions. Quantification of PCR products was performed using the Rotor-Gene Q (Qiagen, Carlsbad, USA). Platinum® SYBR® Green qPCR SuperMix was used to produce fluorescent-labeled PCR products according to the manufacturer's instructions. Primer sets used in this study were previously published and validated (17, 39–41). The β-Actin (*ACTB*) gene was used as a normalizer gene. REST (Relative Expression Software Tool V2.0.21) (42) was applied to calculate the relative gene expression by using the amplification efficiencies from the genes of interest and normalizer *ACTB* gene. The relative expression values obtained by qRT-PCR were transformed to log_2_FC and compared with the log_2_FC expression values determined by the microarray method for the genes selected. Graphs were plotted with GraphPad Prism (v. 6.0).

Supplementary Figure 1 depicts a schematic overview of the experimental design used in this study.

## Results

A genome-wide microarray system to assess differences in the responses of human macrophages (AMs and SMs) to infection with Mtb clinical isolates UT127 and UT205 that were used in this study. Genomic and transcriptomic differences between these isolates have been previously described (24). The aim of this study was to identify the host macrophage early gene expression in response to Mtb infection, and how Mtb affects the transcriptional response of macrophages from healthy individuals and TB patients. Furthermore, we hypothesized that the profiles of gene expression induced by Mtb differ among the different populations of human tissue macrophages analyzed.

### Global Transcriptome Profiles

Hierarchical clustering (*hclust*) was performed to estimate the sample relations based on 20,310 normalized and filtered genes. These genes were selected based on a large coefficient of variance (mean/standard variance > 0.1) as a first descriptive step to compare transcriptomic profiles among the different groups and visualize the relative contributions of stimulus type (Supplementary Figure 2). The samples were grouped according to cell type (AMs and SMs), but not according to their origin (CT or TB). Likewise, it was observed that the samples were grouped according to the type of stimulus (infected with Mtb or not infected). However, we found that two RNA samples (represented as numbers 77 and 54, Supplementary Figure 2), which belonged to the AMCT and AMTB groups infected with Mtb, were grouped as uninfected samples. These samples were considered as outliers and were removed from the analysis in the subsequent steps. In contrast, although SMs constitute a separate group from AMs, they are not discriminated between NI samples and samples infected with Mtb. This grouping was not discriminated between the infection with the clinical isolates UT127 and UT205, suggesting a lower response to Mtb infection compared to AMs. A sample of RNA from SMs infected with UT127 (represented as numbers 15, Supplementary Figure 2) was removed from the analysis through preprocessing and quality. The final analyses were performed with 35 RNA samples.

**Figure 1:**
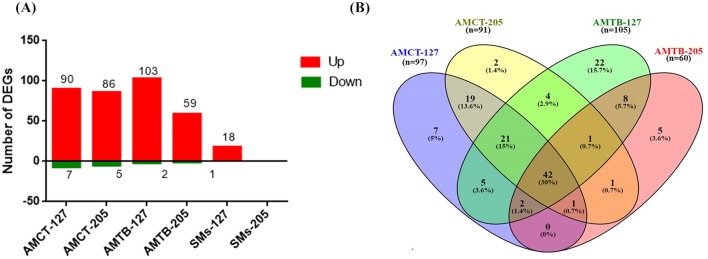
Number and comparison of differentially expressed genes (DEGs) in human tissue macrophages infected with *M. tuberculosis*. **(A)** The amount of upregulated (red) and downregulated (green) DEGs (1.5 log2-fold, *p* ≤ 0.05) in alveolar macrophages (AMs) from control (AMCT, *n* = 4), TB patients (AMTB, *n* = 4), and splenic macrophages (SMs, *n* = 5) infected with *M. tuberculosis* clinical strains UT127 or UT205. **(B)** Venn diagram comparing DEGs in AMCTs and AMTBs infected with *M. tuberculosis* strains UT127 and UT205.

Based on this clustering, a statistical analysis was carried out to identify DEGs in each group. DEGs were selected considering the non-infected samples in each group. In response to infection with Mtb UT127, AMCT exhibited 97 DEGs; AMTB, 105 DEGs; and SMs, 18 DEGs. In response to infection with UT205, AMCTs exhibited 91 DEGs and AMTBs exhibited 60 DEGs, while SMs did not show DEGs (Figure 1A). A full list of DEGs for each group is provided in Supplementary Table 1.

### Transcriptomic Response of AMCTs and AMTBs to Infection With Mtb UT127 and UT205

To identify changes in transcriptional profiles and differential gene expression levels between Mtb-infected human AMs, we compared the transcriptomes of AMCTs and AMTBs infected with clinical isolates UT127 and UT205 (Figure 1B). Supplementary Table 2 shows the list of unique and shared genes in each comparison.

The comparison of the transcriptional profiles showed that most of the DEGs were shared among all the groups, while few genes were associated with specific response to the infection with the Mtb clinical isolates (Figure 1B). In all comparisons, the common genes were expressed in the same direction. In terms of the magnitude of expression, Log_2_FC values were higher in response to infection with UT127 in both AMCTs and AMTBs, whereas in response to infection with UT205, the Log_2_FC values were lower (Supplementary Table 2).

Initially, we searched for the common DEGs of macrophages infected with Mtb, independent of the type of macrophage or Mtb isolate, defining a core macrophage transcriptome. The pairwise analysis revealed 67 genes (47.7%) that were shared among three or four groups and represented a global macrophage transcriptional response to Mtb infection. Of these, 42 genes (30%) were common in all groups and upregulated, except the *PDK4* gene, which was downregulated. Twenty-one genes (15%) were common among AMCT-127, AMCT-205, and AMTB-127, of which 20 genes were upregulated, and one gene (*GPR34*) was downregulated. Two upregulated genes (1.4%) (*RSAD2* and *SLAMF1*) were common among AMCT-127, AMTB-127, and AMTB-205, and the *MARCKS* gene (0.7%) was common among AMCT-127, AMCT-205, AMTB-205, and *CXCL11* (0.7%), which was common between AMCT-205, AMTB-127, and AMTB-205, respectively (Figure 1B, Supplementary Table 2).

**Figure 2:**
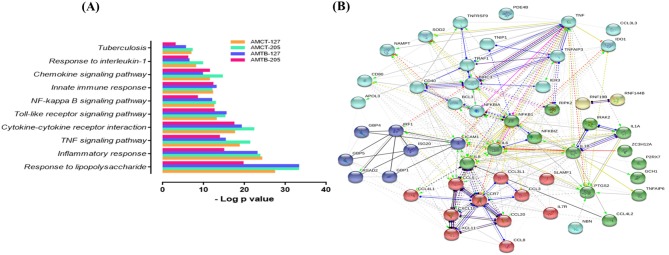
Global response of macrophages to infection with *M. tuberculosis*. **(A)** Functional categories (GO and KEGG pathways) were significantly enriched (FDR ≤ 0.01) among DEGs expressed in alveolar macrophages (AMs) from control subjects (AMCTs) and TB patients (AMCTs) infected with *M. tuberculosis* UT127 (AMCT-127, AMTB-127) or UT205 (AMCT-205, AMTB-205). **(B)** Functional protein:protein association network (by GO and KEGG) of 67 correlated genes that were shared among AMCTs and AMTBs infected with *M. tuberculosis* and representing a global macrophage transcriptional response to *M. tuberculosis* infection.

As expected, many upregulated genes present in the common transcriptome of macrophages that respond strongly to Mtb infection are involved in the activation and recruitment of antimicrobial mechanisms relevant to TB pathophysiology. Genes encoding cytokines, such as *IL1B, IL6, TNF*, and *CCL5*, and chemokines such as *CCL20, CCL4L1, CCL3, CCL3L1, CCL4L1, CCL4L2*, and *CCL8*, are associated with recruitment of monocytes and macrophages during granuloma formation (43, 44). In addition, *CXCL10* and *CXCL8* were upregulated; both being useful markers for monitoring treatment in adults with active TB (45, 46) and strong chemoattractants of T cells, monocytes, and neutrophils (47, 48). Several IFN-inducible GTPases (GBPs), including *GBP1, GBP4*, and *GBP5*, IFN-stimulated genes (ISGs), such as *ISG20, RSAD2*, and *IRF1* (interferon-regulatory factors 1), and pro-apoptotic genes (*PTGS2*) were also induced. Only two genes (*PDK4* and *GPR34*) with a deficiency in mice were associated with an altered immune response in TB (49, 50) and downregulated. To obtain more information on the functional organization of the genes, they were subjected to functional enrichment and network interaction analysis (Figure 2). Functional categories (GO) and pathways (KEGG) associated with Mtb infection in AMCTs and AMTBs, responding to a robust inflammatory response to lipopolysaccharide, the TNF signaling pathway, cytokines and chemokines, activation of toll-like receptors (TLRs) and the NF-kappa B signaling pathway, and TB, are relevant (Figure 2A). The interaction network (Figure 2B) showed nodes of genes significantly associated with the functional categories described in Figure 2A. Interestingly, the level of significance of the functional categories enriched in AMTBs infected with Mtb UT205 was lower than that in the rest of the groups.

### Cell-Type-Specific Response to *M. tuberculosis* Infection

**Figure 3:**
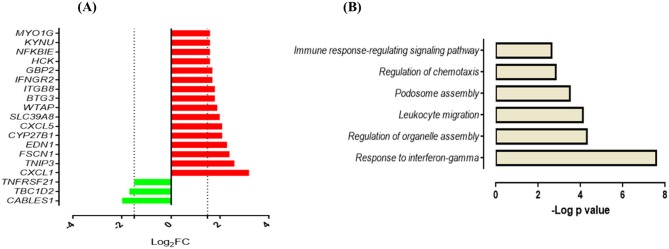
Response of AMCTs to infection with *M. tuberculosis* **(A)**. List of common upregulated (red) and downregulated (green) DEGs (*n* = 19) expressed in AMCTs infected with UT127 and UT205. **(B)**. Functional categories of biological processes by GO and KEGG pathways were significantly enriched.

Next, we compared the response of AMCTs and AMTBs to infection with the clinical isolates of Mtb to better differentiate the effect of Mtb infection in AMTBs. In general, the transcriptome of AMCTs in response to infection with both strains, UT127 and UT205, was similar; however, a lower response (few genes expressed exclusively) was induced with clinical isolate UT205. Thus, 19 genes (13.6%) were commonly expressed in AMCTs infected with UT127 and UT205 (Figure 1B, Supplementary Table 2), of which 16 genes were upregulated and encoded chemokines, such as *CXCL1* and *CXCL5*, Src family tyrosine kinase (*HCK*), tryptophan metabolism enzymes such as kynureninase (*KYNU*), and IFN-inducible GTPases such as *GBP2* and interferon gamma receptors such as *IFNGR2* (Figure 3A). Only three genes, *CABLES1, TBC1D2*, and *TNFRSF2*, without a known function during Mtb infection were downregulated (Figure 3A).

Functional analysis of the upregulated genes showed enrichment in biological processes associated with the gamma interferon (IFN-γ) response (*CYP27B1, GBP2, IFNGR2*), regulation of organelle assembly (*EDN1, HCK, TBC1D2, FSCN1*), migration of leukocytes (*EDN1, CXCL1, HCK, CXCL5, MYO1G*), podosome assembly (*HCK, FSCN1*), regulation of chemotaxis (*CXCL1* and *CXCL5*), and immune response-regulating signaling pathway (*TNFRSF21, MYO1G, TNIP3*) (Figure 3B). In contrast, seven genes (5%) were differentially expressed in response to infection with clinical isolate, UT127 (Figure 1B, Supplementary Table 2), of which five genes were upregulated, including *FAM129A, GJB2*, and *ZSWIM4*, and signaling receptors, such as *ADORA2A*, and prostaglandin E receptor 4 (*PTGER4*), were significantly enriched in a single functional category, such as negative regulation of inflammatory response (*p* = 5.75 × 10^−4^). Only the *HES2* gene (hes family bHLH transcription factor 2), associated with the metabolism of retinoic acid (51, 52), and *DHRS3* (dehydrogenase/reductase) were downregulated.

Conversely, infection of AMCTs with UT205 resulted in the upregulation of only two genes (1.4%). The *G0S2* gene function inhibits the lipolytic enzyme adipose triglyceride lipase (ATGL) and is involved in cellular proliferation and differentiation (53). The *IL4I1* gene encodes an immunosuppressive enzyme (L-phenylalanine oxidase) that inhibits T-cell proliferation via H_2_O_2_ production (54) and is highly expressed in macrophages and DCs of granulomas from sarcoidosis and TB (55). In the present study, no significant functional categories or association networks were observed using the bioinformatics tools.

**Figure 4:**
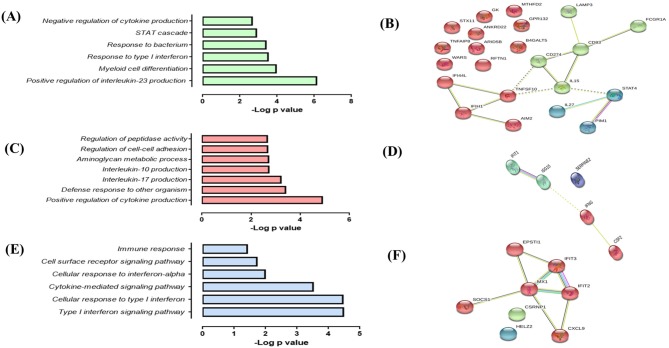
Response of alveolar macrophages (AMs) from TB patients (AMTBs) to infection with *M. tuberculosis* **(A,C,E)**. Functional categories (biological process by GO and KEGG) in response unique to infection with UT127 **(A)**, UT205 **(C)**, or common DEGs of AMTBs upon infection with *M. tuberculosis*. **(C)** Functional association networks of protein–protein interactions were analyzed using String software **(B,D,F)**. Association of upregulated DEGs (*n* = 22) in response to infection with *M. tuberculosis* UT127 **(B)**, upregulated DEGs (*n* = 5) in response to infection with *M. tuberculosis* UT205, and commonly upregulated genes (*n* = 8) upon infection with *M. tuberculosis* UT127 or UT205.

The response of AMTBs to Mtb infection indicated a higher induction of genes with the clinical isolate, UT127, compared with the transcriptional response induced by UT205. Thus, 22 genes (15.7%) were exclusively upregulated in response to infection with UT127 (Figure 1B, Supplementary Table 2), including receptors (*AIM2, CD274, CD83, FCGR1A*, and *GPR132*), enzymes (*B4GALT5, GK, MTHFD2, PIM1*, and *WARS*), cytokines (*IL15, IL27, TNFAIP8*, and *TNFSF10*), IFN-inducible genes (*IFI44L* and *IFIH1*), protein-trafficking (*LAMP3, ANKRD22, RFTN1*, and *STX11*), and transcription factors (*ARID5B* and *STAT4*). These genes were enriched in biological functional categories, including positive regulation of cytokine production (*IL27, CD274, IL15, IFIH1, CD83*, and *AIM2*), defense response to other organisms (*IFI44L, IL27, IL15, IFIH1*, and *AIM2*), interleukin-17 production (*RFTN1, IL15*), interleukin-10 production (*CD274, CD83*), aminoglycan metabolic process (*IL15, PIM1, B4GALT5*), regulation of cell–cell adhesion (*IL27, CD274, IL15, CD83*), and regulation of peptidase activity (*TNFAIP8, LAMP3, TNFSF10, AIM2*) (Figure 4A). The interaction network (Figure 4B) features nodes of genes (indicated in different colors) significantly associated with the functional categories described in Figure 4A.

In response to infection with UT205, AMTBs induced the expression of five genes (3.6%) (Figure 1B, Supplementary Table 2) associated with positive regulation of interleukin-23 production (*CSF2, IFNG*), myeloid cell differentiation (*CSF2, IFNG, ISG15, SERPINE2*), response to type I IFN, and negative regulation of cytokine production (*IFIT1, ISG15*) (Figures 4C,D).

Eight upregulated genes (5.7%) were common to infection of AMTBs with both clinical isolates of Mtb (Figure 1B, Supplementary Table 2) and were mainly associated with functional categories of ISGs (Figures 4E,F), including *IFIT2, IFIT3, MX1, HELZ2, EPSTI1, CSRNP1*, and *CXCL9*, which are induced by IFN-γ and participate in the recruitment of activated CD4 T cells and monocytes (56). Of interest, the *SOCS1* gene (suppressor of cytokine signaling 1) was differentially expressed by AMTBs in response to Mtb. SOCS1 modulates immunity and inflammation, providing negative feedback to suppress cytokine activities, such as those dependent on IFN-γ (57). Recent studies have indicated that SOCS1 expression is higher in patients with active TB than in healthy subjects (58, 59). SOCS1 has also been reported to be associated with caseous necrosis in granulomas from patients with TB lymphadenitis (59).

### SMs Display a Distinct and Attenuated Response to Infection With Mtb Clinical Isolates UT127 and UT205

**Figure 5:**
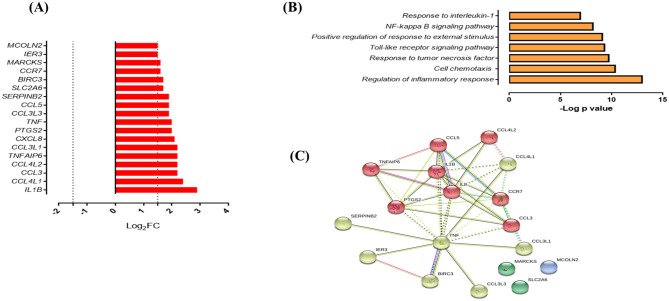
Response of SMs to infection with *M. tuberculosis*. **(A)** List of upregulated genes (red) expressed (log2 fold) in response to infection with *M. tuberculosis* UT127 (*n* = 18). **(B)** Functional categories (biological process by GO and KEGG) in response to infection with *M. tuberculosis* UT127. **(C)** Functional protein:protein association network of 18 genes upregulated in response to infection with UT127 as defined by the String database.

In response to infection with Mtb UT127, SMs expressed 18 upregulated genes (Figure 5A), whereas in response to infection with UT205, SMs did not exhibit DEGs.

Functional analysis of these genes was mainly associated with the response to chemotaxis cells (*CCL3, CCL3L1, CCL3L3, CCL4L1, CCL4L2, CCL5, CCR7, CXCL8*, and *IL1B*), inflammatory response, and response to tumor necrosis factor (*BIRC3, PTGS2, TNF*, and *TNFAIP6*), response to interleukin-1 (*CXCL8, CCL4L1, CCL3L3, CCL3*, and *CCL5*), and regulation of response to external stimulus (*SERPINB2, IER3, BIRC3, PTGS2*, and *TNFAIP6*) (Figure 5B). The interaction network (Figure 5C) features nodes of genes significantly associated with the functional categories described in Figure 5B. Of these genes expressed by SMs in response to infection with UT127, 17 genes were common to AMs response to infection with Mtb (although with a lower fold-change compared to AM), whereas only one gene (*SERPINB2*) was expressed exclusively by SMs (data not shown). Although SMs featured few functional categories in response to infection with UT127, these were common to the functional categories found in AMs and therefore reflect the response of macrophages to Mtb infection.

### Canonical Pathways and Network Analysis of DEGs

Canonical pathways and networks along with molecular and cellular functions were significantly identified (*p* < 0.01) and *Z* score that predicted activation (≥2) or repression (≤ 2) in AMCTs, AMTBs, and SMs infected with Mtb UT127 and UT205. A complete list of all the enriched pathways for each condition is shown in Supplementary Table 3.

**Figure 6:**
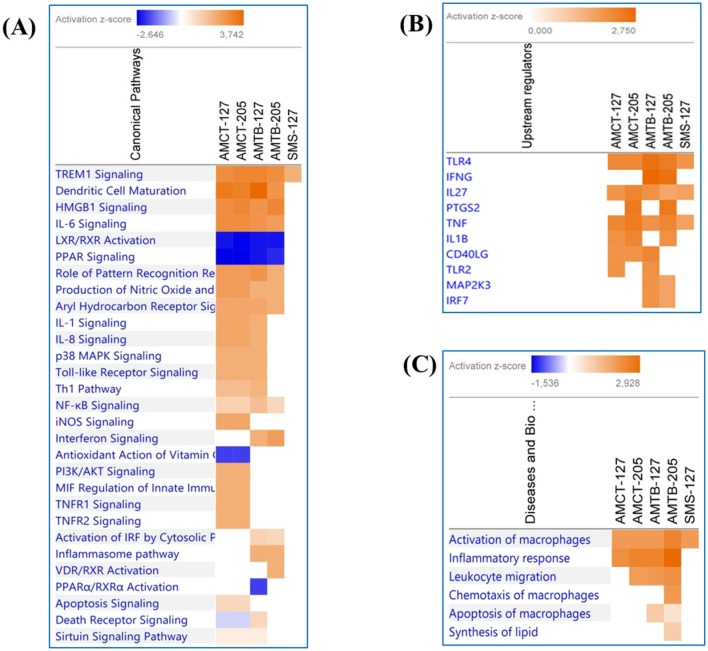
Comparison of significant canonical pathways and upstream regulators in AMCTs, AMTBs, and SMs infected with *M. tuberculosis* identified by the Ingenuity Pathway Analysis (IPA). **(A)** Top enriched canonical pathways, **(B)** upstream regulators, and **(C)** diseases and biological functions in AMCTs, AMTBs, and SMs infected with *M. tuberculosis*. Pathways were ranked according to the enrichment score (Fisher's exact test *p* value ≤ 0.05), and the *Z* score that predicts activation (≥2) or repression (≤ 2).

A comparative analysis was aimed at visualizing the canonical pathways and upstream regulators across all conditions (Figure 6). Figure 6A displays the canonical pathways enriched in all groups, which were associated mainly with immune response reacting to Mtb infection. Most of the pathways were activated (*Z* score ≥2) in AMCTs and AMTBs; however “iNOS signaling,” “TNFR1 signaling,” “TNFR2 signaling,” “PI3K/AKT signaling,” “MIF regulation of innate immunity,” and “Apoptosis signaling” were only activated in AMCT-127 and AMCT-205, while “Interferon signaling” and “Activation of IRF cytosolic pattern recognition receptors” were pathways activated exclusively in AMTBs in response to infection with UT127 and UT205. All groups, including SMs, activated the canonical “TREM1 signaling,” while “Inflammasome pathway” was only activated in AMTB-127. In contrast, “LXR/RXR Activation” and “PPAR Signaling” were inhibited (*Z* score ≤ 2).

The upstream analysis revealed the activation of molecules, receptors, and transcriptional regulators of which prediction is based on DEGs in each group of macrophages (AMCTs, AMTBs, and SMs) in response to Mtb infection, which can be important during TB (Figure 6B). Thus, *TLR4, IL27*, and *TNF* were activated across all groups; *IFNG* was exclusively activated by AMTB-127 and AMTB-205; *PTGS2* was activated in AMCTs and AMTBs in response to infection with Mtb UT205, whereas *TLR2* was only activated in response to Mtb UT127. The biological functions in AMCTs, AMTBs, and SMs infected with Mtb UT127 and UT205 were mainly associated with macrophage activation and inflammatory response (Figure 6C).

**Figure 7:**
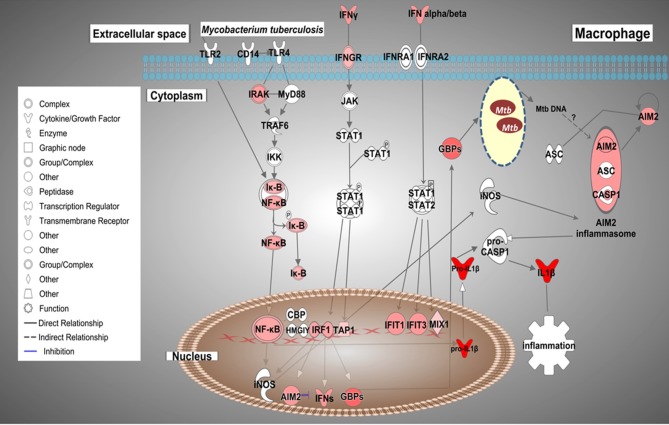
Possible cross-talk of IFNs and the AIM2 inflammasome pathways in AMs from TB patients (AMTB) modulated by *M. tuberculosis*. Upon recognition of mycobacterial components via TLR signaling and subsequent activation of the transcription factor, NF-kB, IFNs (type I and II) are expressed in AMs. IFNs bind to their specific receptors and activate the signaling pathway responsible for DNA transcription and expression of ISGs, including IRF1 as well as the inflammasome components, AIM2. IRF1 promotes the expression of GBPs and IFNs, mainly IFN-γ. GBPs permeabilize the membrane of Mtb, resulting in the release of bacterial DNA. *M. tuberculosis* cytosolic DNA is sensed by the AIM2 inflammasome. IRFs also stimulate the expression of antimicrobial genes, such as iNOS, which potentiates the activation of the AIM2 inflammasome. AIM2 promotes the maturation of pro-IL-1β to IL-1β, which has pro-inflammatory activity. The sustained production of IL-1β is based on AIM2-suppressing type I IFNs (see Discussion). Genes significantly upregulated are shown in red. The intensity of red corresponds to an increase in fold-change levels of AMTB infected with *M. tuberculosis* compared to control. Genes in white did not exhibit significant changes in expression. The network was generated with IPA (Fisher test, *p* value ≤ 0.05).

Based on the biological importance of the AMTB response in the immunopathology of TB, we constructed a model based on the canonical pathways activated in response to infection with Mtb clinical isolates (Figure 7). This model showed the enrichment of genes in “Interferon signaling” and the “Inflammasome pathway,” suggesting cross-talk of IFNs and *AIM2* inflammasome pathways during active TB response.

### Technical Validation of Differential Expression by Quantitative Real-Time PCR

The results garnered by microarray were validated through qRT-PCR analysis evaluating the expression levels of four genes of interest (*IL1B, IL8, TNF*, and *TNFAIP6*), while the *ACTB* gene was used as the internal control. The relative expression showed that these genes were significantly upregulated (Supplementary Figure 3). The relative expression level of each gene was transformed into Log_2_FC and compared with the expression values obtained by microarray, and the results from the two techniques were complementary (Supplementary Figure 4).

## Discussion

Macrophages are considered the first line of defense against respiratory pathogens and play essential roles in the pathophysiological response to Mtb infection in humans (60). AMs constitute the initial niche in which a proinflammatory response is triggered upon Mtb infection. However, under certain circumstances, Mtb can be disseminated to other organs and tissues by interacting with alternative macrophage populations. Remarkably, the response to Mtb infection by AMs from TB patients is poorly known, and very little is known about the transcriptomic response of extrapulmonary macrophages to Mtb infection.

### AMs of TB Patients Respond to Infection With Virulent Mtb Clinical Isolates, With Upregulation of Genes Associated With IFN Signaling Pathways

Compared to AMCTs, infection of AMTBs with Mtb induced the expression of ISGs, by upregulating IFIT family genes and GBPs. In addition, essential ISGs, including *IRF1* as well as the inflammasome component *AIM2*, were upregulated in AMTBs. *IRF1* is a transcription factor that regulates the expression of GBPs, a family of large GTPases with antimicrobial functions by the destruction of vacuoles containing pathogens, inducing inflammasome assembly and autophagy (61). GBPs limit cytosolic bacteria and expose them to cytosolic PRRs and PAMPs, such as dsDNA (62). In murine macrophages exogenously treated with IFN-γ and infected with *Mycobacterium bovis*, BCG attenuation of *Gbp1, Gbp5*, or *Gbp7* expression reversed the anti-mycobacterial effect of IFN-γ (63), demonstrating the importance of GBPs in mycobacterial infection. The role of IFIT family during Mtb infection remains unknown. In contrast to our observation of *IFIT1* upregulation in AMs, a recent study showed that the *IFIT1* gene was one of the most downregulated genes in murine BMDMs infected with Mtb H37Rv for 4 days (64). Although no anti-mycobacterial effects were attributed to *IFIT1*, an early anti- or pro-mycobacterial effect depending on Mtb strain or virulence and macrophage species cannot be ruled out.

Cytosolic dsDNA sensors, such as cGAS or AIM2, recognize mycobacterial DNA in the cytosol (65), and this recognition depends on the phagosomal rupture induced by the mycobacterial ESX-1 transport system (66–68). Interestingly, two recent reports in bovine AMs infected with *M. bovis* or Mtb H37Rv have also highlighted the role of TLR signaling and nucleic acid recognition in the innate immune response to mycobacterial antigens (69, 70), indicating a clear parallel between the human and bovine AM response to mycobacterial infections.

Notably, the AIM2 inflammasome is involved in the activation of mouse macrophages during infection with a virulent *M. bovis* strain presumably translocated to the cytosol (71). Macrophages from AIM2-deficient mice were highly susceptible to intratracheal infection with Mtb, and this deficiency resulted in severe inhibition of the AIM2 inflammasome, associated with defective IL1 and IL18 production and impaired Th1 responses (72).

The IL1 family of cytokines, including IL-1β, possesses potent pro-inflammatory activities (73) and is responsible for host defense against mycobacteria (74–76). We previously reported the baseline transcriptome of uninfected bystander AMTBs (26), where the *IL1B* gene was downregulated. However, in the present study, we showed that after infection with Mtb, AMTBs upregulated the *IL1B* gene. Consequently, these results suggest that the expression of *IL1B* may depend on the AIM2 signaling pathway in response to Mtb infection, mainly based on the clinical isolate, UT127. Thus, we speculate that Mtb or Mtb DNA may translocate to the cytosol and activate the AIM2 inflammasome. The mechanisms of Mtb DNA recognition in the cytosol by the AIM2 inflammasome remain unexplored.

Previous evidence indicates that AIM2 might be involved in the signaling pathway responsible for the suppression of type I IFNs (IFN-β) (3, 77). Mtb infection induces IFN-β expression, resulting in suppression of IL-1β production by mouse macrophages and dendritic cells (76) as well as human MDMs (77). As type I IFNs contribute to impaired host resistance to Mtb in mice (78–80), it may be possible that *AIM2* participates in two signaling pathways: mediating the inflammasome-dependent processing of the IL1 family of cytokines, and the second mediating activation of pathways that may sustain the production of IL-1β by suppressing type I IFNs. Our findings suggest a critical role for AIM2 in Mtb infection. Based on these findings, we propose a model of cross-talk of ISGs and AIM2 inflammasome pathways/*IL1B*, which can be modulated by mycobacterial DNA inside infected AMs during TB (Figure 7).

Several studies support the finding that IFN-β promotes Mtb infection, while IFN-γ may exert a protective effect against Mtb (81, 82). Given that ISGs are induced by both type I IFN and type II IFN, our data suggest that both signaling pathways might be modulated in AMs during TB disease. These observations indicate that host control of Mtb and pathology may depend on the relative balance of type I and type II IFN functions.

*FCGR1A* was also expressed upon infection with Mtb. *FCGR1A* expression is induced by IFN-γ in monocytes and macrophages while also playing a critical role in the clearance of immune complexes and antibody-dependent cytotoxicity (83). Recently, it was determined that a subset of transcripts enriched for IgG receptors was present in the whole blood of symptomatic TB patients and individuals with subclinical disease, suggesting an early cellular response to antigen/antibody complexes during TB disease in HIV-infected individuals (84). Additionally, the higher expression levels of FCGR1A in TB patients regardless of HIV status or genetic background have been considered a remarkable and consistent classifier of active disease (85). *FCGR1A*, in combination with other genes, has evident discriminatory power between TB and latent TB infection (86, 87). Functional correlations of pathogenesis-driven gene expression signatures in TB indicate that *FCGR1A* is associated with apoptotic and pro-inflammatory regulators (88). Previous findings in South African TB patients showed a significant reduction in *FCGR1A* expression following treatment for TB (87). Therefore, we suggest that the expression of *FCGR1A* in AMTBs could indicate the likely importance of this receptor in TB pathogenesis and reinforces its possible use as a biomarker. However, these findings require further validation.

Additionally, the transcriptional signature observed in AMTBs in response to Mtb infection was associated with myeloid cell inflammation and TREM1 signaling as a top-scoring canonical pathway across all macrophage populations evaluated in this study (AMCTs, AMTBs, and SMs) (Figure 6A). Our findings are in line with recently published reports (15), indicating TREM1 as a top inflammatory signaling pathway playing a critical role in controlling inflammatory processes.

Overall, the response of AMTBs to Mtb infection is dominated by IFN-signaling pathways and associated genes (GBPs, ISGs, and IFITs), the implications of which are still under review. In addition to the IFN-signaling signature, several vital pathways in active TB, such as the inflammasome (*AIM2*), FC pathway receptor (*FCGR1A*), and myeloid inflammatory pathway (*TREM1*), were upregulated. However, the specific order in which these processes occur during the earliest stages of the disease are yet to be elucidated. Understanding the early host–pathogen interaction at the onset of infection and active disease may provide insights that could lead to novel approaches to diagnose and manage subclinical disease. To the best of our knowledge, no study has reported such signaling pathways in AMTBs so far.

### SMs Showed an Attenuated Gene Expression Profile in Response to Infection With Mtb Clinical Isolates

In situations of immunosuppression, Mtb spreads to other organs and tissues, generating extrapulmonary forms of TB. Mtb DNA has been amplified from biopsies of organs belonging to individuals whose death was not because of TB (13), suggesting that even in conditions of latent TB, the bacillus could spread to different organs, and the biology of the infection is virtually unknown.

The analysis of the mRNA profiles of SMs infected with the clinical isolates showed a low level of differential gene expression with UT127 infection, and no DEGs were found with clinical isolate UT205 compared with non-infected SMs. These results suggest critical differences between AMs and SMs in their capacity to respond to Mtb.

Functional analysis of upregulated genes by UT127 was mainly associated with pro-inflammation, which could be sustained with the previous results of our group where non-infected SMs produced high basal levels of IL-10 but low levels of IL-12p70, resembling anti-inflammatory macrophages (31). Interestingly, in conditions mimicking high mycobacterial proliferation, SMs produced large amounts of IL-12p40 but not IL-12p70, resembling pro-inflammatory macrophages (31).

Surprisingly, infection with UT205 did not show any DEGs compared with non-infected SMs. We observed that in AMs, the response to infection with clinical isolate UT205 was attenuated when considering the number of DEGs, the magnitude of expression, *p* values of enrichment in functional analysis, and type of induced immune response compared with the response observed with UT127. Our group has provided evidence that these clinical isolates are phenotypically different at the level of cell death, cytokine production, growth kinetics upon *in vitro* infection of human tissue macrophages, membrane vesicle secretion upon culture in synthetic medium, and in their transcriptional strategy to adapt to the stressful conditions imposed by a carbon-poor medium (23, 24).

Recent evidence has shown that different macrophage populations display a distinct response to Mtb infection. For example, in mouse models, AMs are less capable of controlling infection compared to lung interstitial macrophages (89). In addition, SMs from infected mice displayed attenuated metabolomic profiling in response to Mtb infection compared to AMs (90). This difference may be sustained, at least in part, based on the observation that SMs and AMs display distinct transcriptomic profiles (91, 92). Therefore, our results extend and reinforce the fact that a particular response depends on the macrophage population involved, and, importantly, the attenuated response of SMs compared to AMs, which may suggest a better adaptation of Mtb to lung infection compared to extrapulmonary infections.

### The Differential Transcriptomic Response to UT127 and UT205 Suggests Differences in Virulence

Throughout the entire study, we analyzed the impact of the infection by the two clinical isolates, UT127 and UT205, on the immune response of AMs and SMs. In general, both clinical isolates exhibited overlapping changes in the expression of specific genes and pathways, probably reflecting their genome similarity. However, a marked specific response was also observed for each clinical isolate. Interestingly, UT205 induced a low or no transcriptional response in AMCTs, AMTBs, and SMs, compared to the response induced by UT127. Therefore, we suggest that the low immune response observed after AM infection with clinical isolate UT205 and the non-expression of genes seen in SMs infected with UT205 could be partially based on the immune evasion pathways activated by a virulence strain to avoid being sensed by macrophages.

Both clinical isolates not only differentially induced the expression of ISGs via activating type I and type II IFN signaling pathways, differences were also observed in *Z* score values through the enrichment of pathways in IPA between clinical isolates UT127 and UT205 (Supplementary Table 3). We speculate that these differences could be due to differential gene expression in different isolates at early stages of infection. Usually, ISGs act as protective immune effectors in host fighting against pathogen infection, but some of them can also be exploited by pathogens to negatively regulate the immune response and help them survive within the host (93, 94). Therefore, the varied, and sometimes paradoxical, effects of IFNs on Mtb infection can be caused by different types and functions of ISGs. These findings provide insight into a combination of host factors and Mtb virulence factors that can influence multiple aspects of the host-immune response during active TB, and which could define the gene expression profiles seen in AMTBs.

Notable limitations of the present study include the small number of samples used, which may be explained by the challenges in obtaining BALs and macrophages from healthy people and TB patients, as well as splenic samples from deceased patients. However, the sample size used was sufficient to achieve statistical significance between the studied groups. The methodological strategy for selection of DEGs, including a robust normalization procedure, cutoffs for FC, and *p* values, allowed us to confidently detect sets of biologically relevant genes. Although microarrays have quantitative and qualitative limitations compared with more robust methods, like RNA-seq, its analytical platform is well-developed for generating reliable results. Whether our observations extend to other cases with active TB from different ancestry and/or infected with clinical strains of the LAM family or other families of Mtb, they need to be replicated in future studies using a larger number of samples. All samples studied were obtained from individuals from the Mestizo population of residents in the metropolitan area of Medellin. Different genetic studies have characterized the ancestry component of this Mestizo population showing mostly a European ancestry (95–97), therefore reducing the potential bias of in transcriptomic responses based on genetic differences among populations with different ancestry (98).

In conclusion, in this pilot study, we have provided, for the first time, evidence indicating that two human tissue macrophage populations show a distinctly differential transcriptional response to infection with circulating clinical strains of *M. tuberculosis*. The observed differences between AMs and SMs illustrate the higher pro-inflammatory capacity of AMs relative to SMs, which may suggest a better Mtb adaptation to the cellular microenvironment of AMs. Of note, AMTB patients express genes previously associated with the intracellular control of the infection as well as dysregulated genes associated with the compromised capability of macrophages to contain Mtb infection. In this sense, the early balance between the anti-mycobacterial activities of macrophages and the capacity of Mtb to adapt to the cellular microenvironment might be essential for defining appropriate strategies to contain Mtb infection. Finally, the use of circulating clinical strains of Mtb, instead of H37Rv, permitted us to observe a distinct transcriptomic response to both isolates. This last observation may partially explain the differences observed in the severity of TB disease and the response to TB treatment in different human populations.

## Data Availability Statement

All data have been submitted to the NCBI gene expression omnibus (GEO) database with accession number GSE139825.

## Ethics Statement

This study was approved by the Ethical Committee of the Faculty of Medicine, Universidad de Antioquia, Colombia, and by the Ethical Committee of Clínica Cardiovascular Santa María, Medellín, Colombia. Written consent was voluntarily signed by all pulmonary TB patients (PTB) and control subjects (CT) before sample acquisition. The patients/participants provided their written informed consent to participate in this study.

## Author Contributions

LL performed the experiments, collected and analyzed the data, and wrote the manuscript. HO collected and analyzed the data. LB designed the study, interpreted the data, and wrote the manuscript.

## Conflict of Interest

The authors declare that the research was conducted in the absence of any commercial or financial relationships that could be construed as a potential conflict of interest.

## Abbreviations

AMs: alveolar macrophages
AMCT: alveolar macrophages from control subjects
AMTB: alveolar macrophages from tuberculosis patients
BAL: bronchoalveolar lavage
DEGs: differentially expressed genes
FC: fold-change
FDR: false discovery rate
IFIT: interferon-induced proteins with tetratricopeptide repeats
ISG: interferon-stimulated genes
GBP: guanylate-binding proteins
MDMs: monocyte-derived macrophages
MOI: multiplicity of infection
MPS: mononuclear phagocytic system
Mtb: *Mycobacterium tuberculosis*
PAMP: pathogen-associated molecular patterns
PRR: pattern recognition receptors
PTB: pulmonary tuberculosis
TB: tuberculosis.
