# Supplementary material for: Human Alveolar and Splenic Macrophage Populations Display a Distinct Transcriptomic Response to Infection With Mycobacterium tuberculosis
Source: Front Immunol. 2020 Apr 21;11:630. doi: 10.3389/fimmu.2020.00630 (PMC7186480; doi:10.3389/fimmu.2020.00630)
Supplement: Supplementary file 1 [file Data_Sheet_1.PDF]

Supplementary Figures

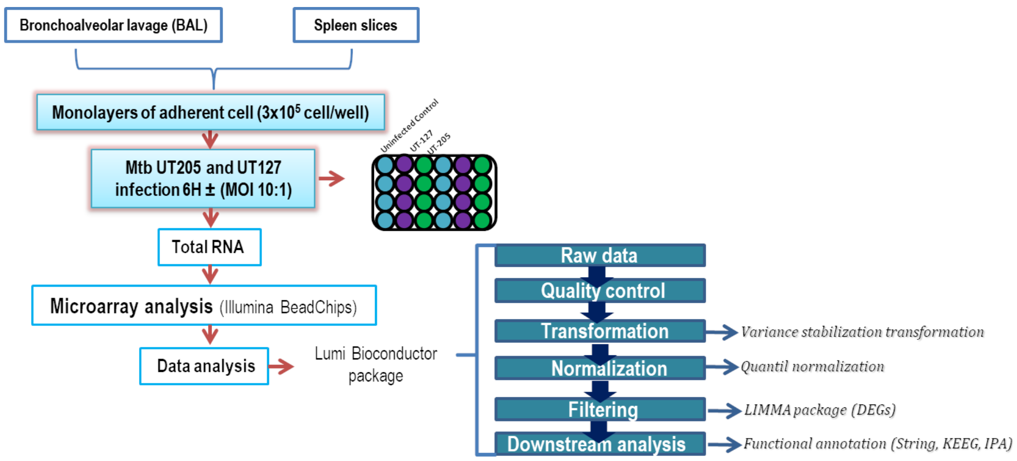

Supplementary Figure 1. Outline of the experimental strategy. Human alveolar macrophages from control subjects (n=4) and tuberculosis (TB) patients (n=4) and splenic macrophages (n=5) were left uninfected (Uninfected controls) or infected for 6 hours (MOI 10:1) with the Colombian clinical isolates UT127 and UT205. At the end of treatment, samples were treated with RNAlater and stored at -80 ^{o}C. After that, the content the replica wells was pooled and total RNA was simultaneously extracted and subjected to Microarray analysis using Illumina BeadChips. Data analysis was conducted with the Lumi Bioconductor package as depicted (further information in the Materials and Methods section).

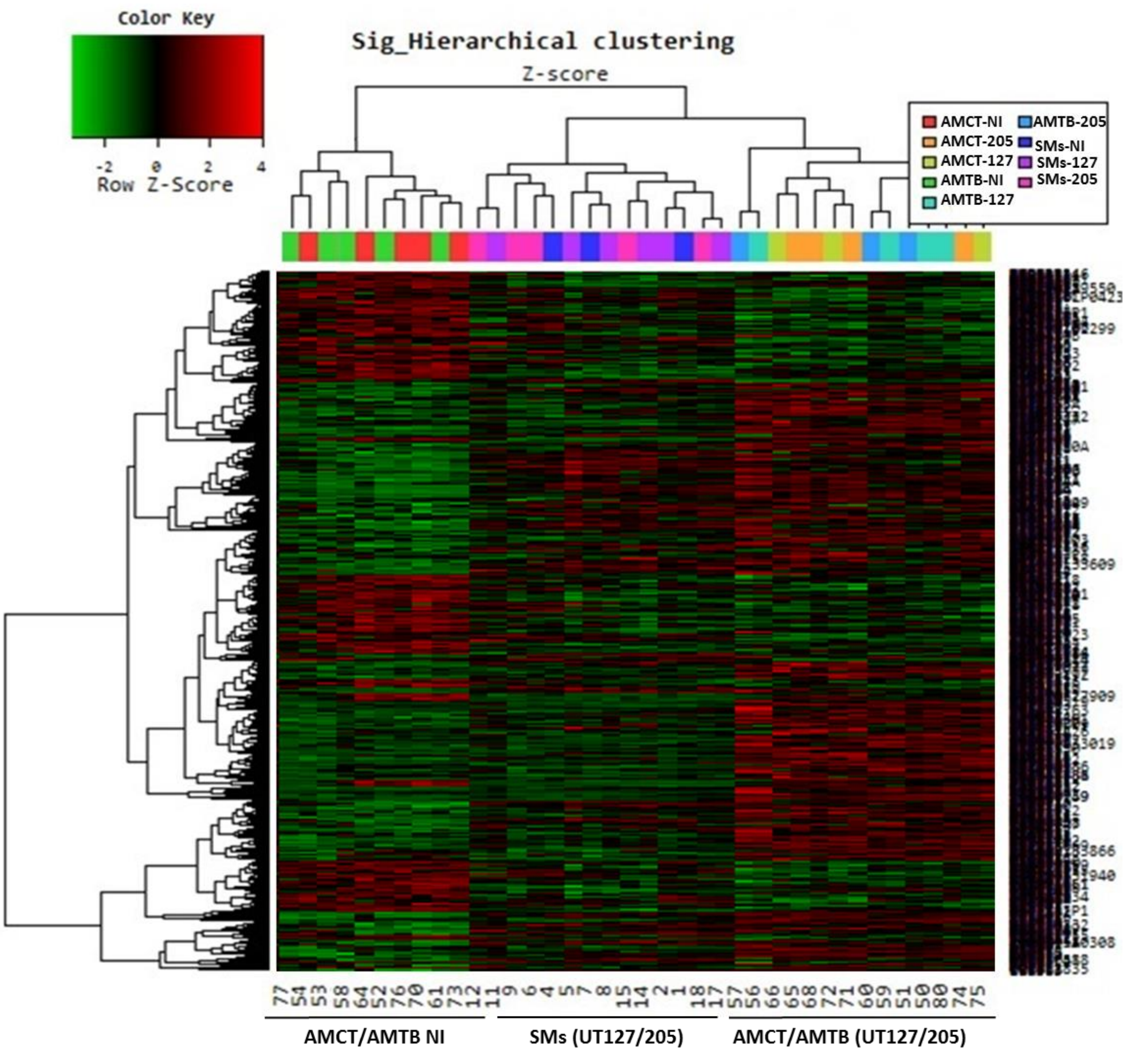

Supplementary Figure 2. Unsupervised hierarchical clustering of gene expression profiles in alveolar macrophages from TB patients (AMTB), healthy control subjects (AMCT) and splenic macrophages (SMs) infected with clinical isolates of *M. tuberculosis* UT127 and UT205. Each row represents a gene; each column shows the expression of 20,037 probe sets expressed by each sample. Red indicates genes that are expressed at higher levels; green indicates genes that are expressed at lower levels and black indicates genes that do not change their expression levels. Two-dimensional hierarchical clustering was applied using a Euclidean Method and Complete Linkage.

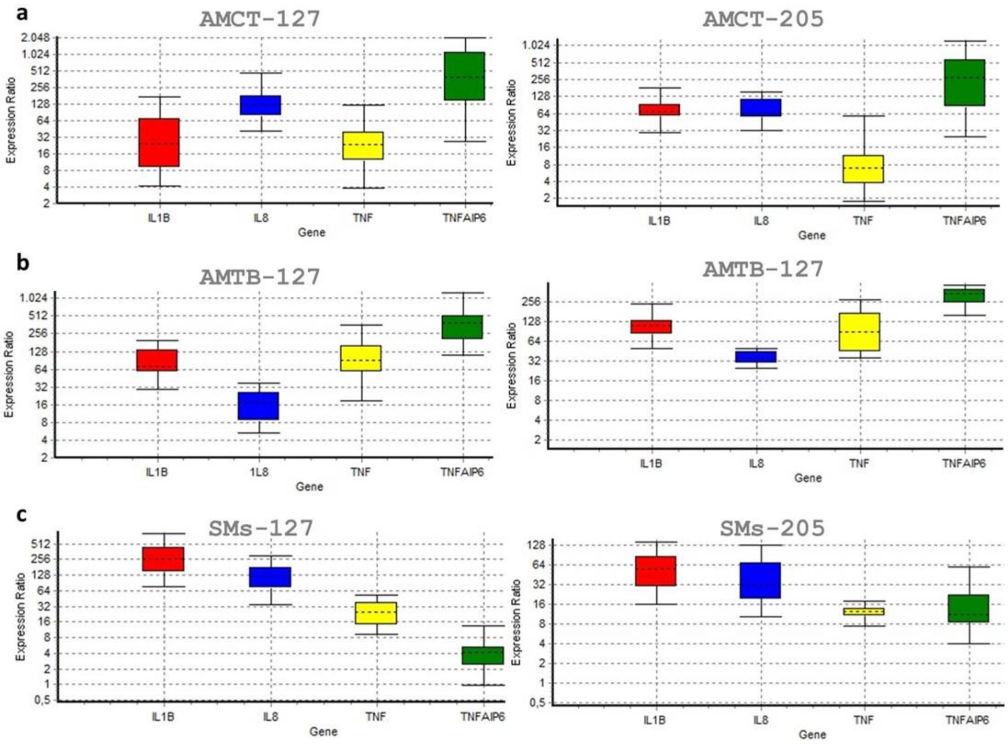

Supplementary Figure 3. Validation of differential expression by quantitative real-time PCR. Relative expression by qRT-PCR of IL1B, IL8, TNF and TNFAIP6 from AMCT, AMTB and SMs infected with Mtb UT127 and UT205 respectively (**A, B** and **C**). The genes of interest were normalized by using the housekeeping gene β-actin. The expression values indicate the fold induction for a duplicate for three different donors.

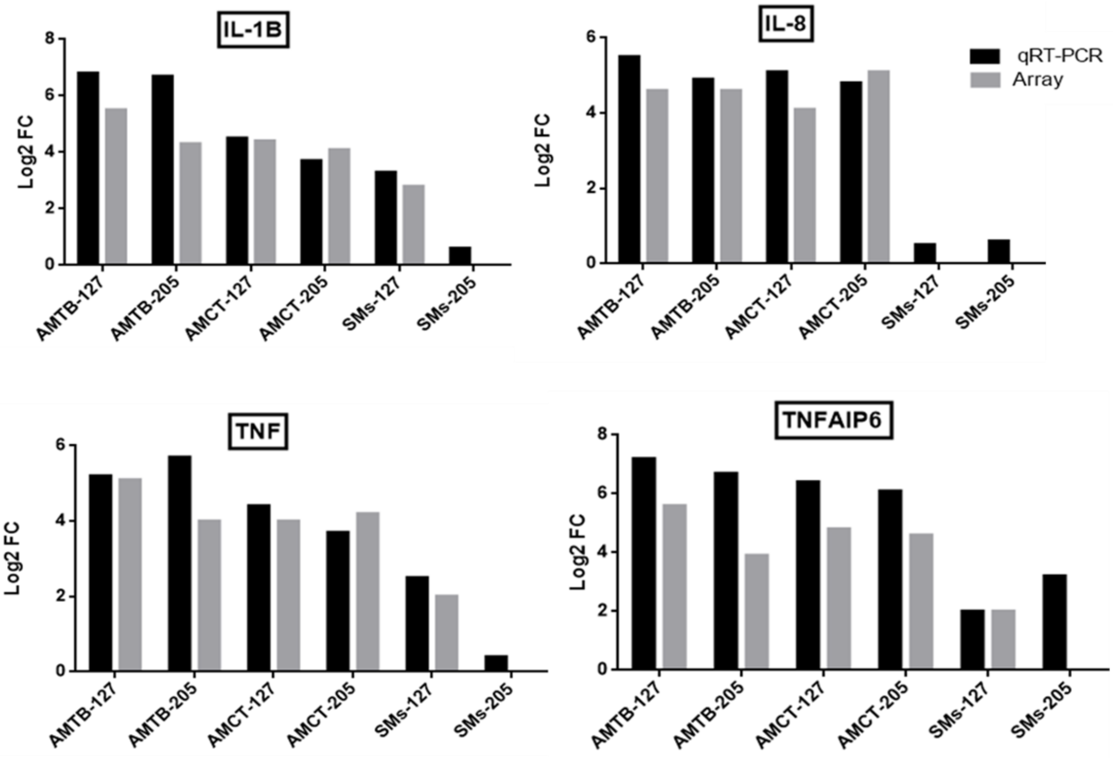

Supplementary Figure 4. Comparison between expression data for both Illumina array and qRT-PCR of the genes of interest. The relative expression levels obtained by qRT-PCR of IL1B, IL8, TNF and TNFAIP6 from AMCT, AMTB and SMs infected with *M. tuberculosis* UT127 and UT205 were transformed to Log2FC and compared with the expression values (Log2FC) obtained by microarray.
