# Supplementary material for: Human Alveolar and Splenic Macrophage Populations Display a Distinct Transcriptomic Response to Infection With Mycobacterium tuberculosis
Source: Front Immunol. 2020 Apr 21;11:630. doi: 10.3389/fimmu.2020.00630 (PMC7186480; doi:10.3389/fimmu.2020.00630)
Supplement: Supplementary file 2 [file Table_1.DOCX]

**Supplementary table 1:** Full list of DEGs for AMCT. AMTB and SMs *in vitro* infected with Mtb UT127 and UT205.

List of DEGs in AMCT infected with Mtb UT127 compared to AMCT-NI

| **Gene** | **Gene Name** | **LogFC** | **P.Value** | **FDR** |
| --- | --- | --- | --- | --- |
| ADORA2A | adenosine A2a receptor | 1.60 | 4.38E-05 | 2.16E-03 |
| APOL3 | apolipoprotein L. 3 | 2.08 | 7.14E-04 | 1.09E-02 |
| BCL3 | B-cell CLL/lymphoma 3 | 1.67 | 2.43E-06 | 5.80E-04 |
| BIRC3 | baculoviral IAP repeat containing 3 | 2.48 | 1.23E-05 | 1.21E-03 |
| BTG1 | B-cell translocation gene 1. anti-proliferative | 1.83 | 4.02E-04 | 7.67E-03 |
| BTG3 | BTG family. member 3 | 1.79 | 3.16E-06 | 6.60E-04 |
| CABLES1 | Cdk5 and Abl enzyme substrate 1 | -1.98 | 5.77E-07 | 3.23E-04 |
| CCL20 | chemokine (C-C motif) ligand 20 | 5.07 | 1.71E-04 | 4.56E-03 |
| CCL3 | chemokine (C-C motif) ligand 3 | 4.13 | 7.91E-06 | 9.92E-04 |
| CCL3L1 | chemokine (C-C motif) ligand 3-like 1 | 4.08 | 5.98E-06 | 8.73E-04 |
| CCL3L3 | chemokine (C-C motif) ligand 3-like 3 | 3.83 | 4.43E-05 | 2.16E-03 |
| CCL4L1 | chemokine (C-C motif) ligand 4-like 1 | 5.11 | 6.37E-05 | 2.68E-03 |
| CCL4L2 | chemokine (C-C motif) ligand 4-like 2 | 4.57 | 7.87E-05 | 2.95E-03 |
| CCL5 | chemokine (C-C motif) ligand 5 | 3.68 | 7.25E-05 | 2.84E-03 |
| CCL8 | chemokine (C-C motif) ligand 8 | 4.11 | 3.85E-06 | 7.08E-04 |
| CCR7 | chemokine (C-C motif) receptor 7 | 2.17 | 2.05E-04 | 5.07E-03 |
| CD40 | CD40 molecule. TNF receptor superfamily member 5 | 1.78 | 1.48E-08 | 7.40E-05 |
| CD80 | CD80 molecule | 2.24 | 1.73E-07 | 2.01E-04 |
| CKB | creatine kinase. brain | 1.84 | 6.81E-04 | 1.06E-02 |
| CXCL1 | chemokine (C-X-C motif) ligand 1 | 3.21 | 6.83E-05 | 2.78E-03 |
| CXCL10 | chemokine (C-X-C motif) ligand 10 | 4.79 | 9.23E-05 | 3.23E-03 |
| CXCL5 | chemokine (C-X-C motif) ligand 5 | 2.07 | 5.10E-04 | 8.92E-03 |
| CXCL8 | chemokine (C-X-C motif) ligand 8 | 4.71 | 6.02E-04 | 9.93E-03 |
| CYP27B1 | cytochrome P450. family 27. subfamily B. polypeptide 1 | 2.13 | 2.09E-06 | 5.40E-04 |
| DENND5A | DENN/MADD domain containing 5A | 1.64 | 3.60E-05 | 1.98E-03 |
| DHRS3 | dehydrogenase/reductase (SDR family) member 3 | -1.53 | 7.56E-07 | 3.55E-04 |
| DUSP5 | dual specificity phosphatase 5 | 1.57 | 9.90E-06 | 1.13E-03 |
| EBI3 | Epstein-Barr virus induced 3 | 1.63 | 6.04E-04 | 9.94E-03 |
| EDN1 | endothelin 1 | 2.32 | 6.31E-08 | 1.58E-04 |
| EHD1 | EH-domain containing 1 | 2.45 | 1.12E-08 | 7.40E-05 |
| FAM129A | family with sequence similarity 129. member A | 1.69 | 2.93E-04 | 6.29E-03 |
| FSCN1 | fascin actin-bundling protein 1 | 2.42 | 2.05E-04 | 5.07E-03 |
| GBP1 | guanylate binding protein 1. interferon-inducible | 3.37 | 7.05E-06 | 9.56E-04 |
| GBP1P1 | guanylate binding protein 1. interferon-inducible pseudogene 1 | 1.84 | 4.96E-05 | 2.33E-03 |
| GBP2 | guanylate binding protein 2. interferon-inducible | 1.66 | 7.69E-06 | 9.72E-04 |
| GBP4 | guanylate binding protein 4 | 2.57 | 4.93E-04 | 8.69E-03 |
| GBP5 | guanylate binding protein 5 | 3.56 | 2.06E-04 | 5.08E-03 |
| GCH1 | GTP cyclohydrolase 1 | 2.81 | 3.28E-07 | 2.74E-04 |
| GJB2 | gap junction protein. beta 2. 26kDa | 2.02 | 4.63E-03 | 3.52E-02 |
| GPR34 | G protein-coupled receptor 34 | -1.68 | 1.68E-05 | 1.41E-03 |
| GRAMD1A | GRAM domain containing 1A | 2.45 | 5.65E-06 | 8.50E-04 |
| HCK | HCK proto-oncogene. Src family tyrosine kinase | 1.63 | 1.63E-04 | 4.44E-03 |
| HES2 | hes family bHLH transcription factor 2 | -1.70 | 7.61E-06 | 9.70E-04 |
| ICAM1 | intercellular adhesion molecule 1 | 2.14 | 1.02E-06 | 3.82E-04 |
| IDO1 | indoleamine 2.3-dioxygenase 1 | 2.03 | 2.75E-04 | 6.02E-03 |
| IER3 | immediate early response 3 | 3.31 | 1.08E-05 | 1.15E-03 |
| IFNGR2 | interferon gamma receptor 2 (interferon gamma transducer 1) | 1.74 | 1.19E-06 | 3.92E-04 |
| IL1A | interleukin 1. alpha | 3.07 | 8.46E-05 | 3.05E-03 |
| IL1B | interleukin 1. beta | 4.39 | 2.68E-04 | 5.91E-03 |
| IL23A | interleukin 23. alpha subunit p19 | 1.85 | 5.58E-03 | 3.96E-02 |
| IL6 | interleukin 6 | 4.03 | 1.70E-05 | 1.42E-03 |
| IL7R | interleukin 7 receptor | 3.27 | 2.38E-05 | 1.60E-03 |
| IRAK2 | interleukin-1 receptor-associated kinase 2 | 2.43 | 1.67E-05 | 1.40E-03 |
| IRF1 | interferon regulatory factor 1 | 2.25 | 7.42E-05 | 2.87E-03 |
| ISG20 | interferon stimulated exonuclease gene 20kDa | 1.60 | 1.43E-04 | 4.14E-03 |
| ITGB8 | integrin. beta 8 | 1.83 | 1.82E-06 | 5.09E-04 |
| KYNU | kynureninase | 1.61 | 2.32E-05 | 1.60E-03 |
| MARCKS | myristoylated alanine-rich protein kinase C substrate | 2.13 | 2.89E-04 | 6.22E-03 |
| MCOLN2 | mucolipin 2 | 2.65 | 1.13E-05 | 1.15E-03 |
| MYO1G | myosin IG | 1.59 | 3.40E-04 | 6.94E-03 |
| NAMPT | nicotinamide phosphoribosyltransferase | 2.24 | 3.14E-05 | 1.84E-03 |
| NBN | nibrin | 2.21 | 5.79E-07 | 3.23E-04 |
| NEURL3 | neuralized E3 ubiquitin protein ligase 3 | 1.95 | 2.29E-05 | 1.60E-03 |
| NFKB1 | nuclear factor of kappa light polypeptide gene enhancer in B-cells 1 | 2.23 | 9.78E-07 | 3.78E-04 |
| NFKBIA | nuclear factor of kappa light polypeptide gene enhancer in B-cells inhibitor. alpha | 2.03 | 3.79E-05 | 2.05E-03 |
| NFKBIE | nuclear factor of kappa light polypeptide gene enhancer in B-cells inhibitor. epsilon | 1.65 | 2.01E-04 | 5.01E-03 |
| NFKBIZ | nuclear factor of kappa light polypeptide gene enhancer in B-cells inhibitor. zeta | 2.09 | 3.84E-06 | 7.08E-04 |
| P2RX7 | purinergic receptor P2X. ligand-gated ion channel. 7 | 2.05 | 9.79E-07 | 3.78E-04 |
| PDE4B | phosphodiesterase 4B. cAMP-specific | 2.06 | 8.11E-05 | 3.02E-03 |
| PDK4 | pyruvate dehydrogenase kinase. isozyme 4 | -1.64 | 8.03E-05 | 3.00E-03 |
| PSTPIP2 | proline-serine-threonine phosphatase interacting protein 2 | 2.63 | 1.27E-07 | 2.01E-04 |
| PTGER4 | prostaglandin E receptor 4 (subtype EP4) | 1.51 | 1.39E-05 | 1.25E-03 |
| PTGS2 | prostaglandin-endoperoxide synthase 2 (prostaglandin G/H synthase and cyclooxygenase) | 3.77 | 5.34E-05 | 2.42E-03 |
| RIPK2 | receptor-interacting serine-threonine kinase 2 | 1.86 | 5.50E-09 | 7.40E-05 |
| RNF144B | ring finger protein 144B | 1.86 | 3.93E-06 | 7.08E-04 |
| RNF19B | ring finger protein 19B | 1.60 | 5.71E-06 | 8.50E-04 |
| RSAD2 | radical S-adenosyl methionine domain containing 2 | 1.60 | 4.62E-04 | 8.43E-03 |
| SLAMF1 | signaling lymphocytic activation molecule family member 1 | 2.12 | 1.49E-04 | 4.22E-03 |
| SLAMF7 | SLAM family member 7 | 1.65 | 8.18E-05 | 3.02E-03 |
| SLC25A24 | solute carrier family 25 (mitochondrial carrier; phosphate carrier). member 24 | 2.29 | 1.08E-04 | 3.51E-03 |
| SLC2A6 | solute carrier family 2 (facilitated glucose transporter). member 6 | 3.22 | 1.18E-06 | 3.92E-04 |
| SLC39A8 | solute carrier family 39 (zinc transporter). member 8 | 2.01 | 4.19E-06 | 7.33E-04 |
| SOD2 | superoxide dismutase 2. mitochondrial | 3.16 | 2.06E-06 | 5.40E-04 |
| TAP1 | transporter 1. ATP-binding cassette. sub-family B (MDR/TAP) | 1.89 | 9.90E-06 | 1.13E-03 |
| TBC1D2 | TBC1 domain family. member 2 | -1.67 | 3.92E-05 | 2.05E-03 |
| TMEM194A | transmembrane protein 194A | 1.57 | 5.74E-07 | 3.23E-04 |
| TNF | tumor necrosis factor | 4.01 | 2.70E-06 | 6.06E-04 |
| TNFAIP3 | tumor necrosis factor. alpha-induced protein 3 | 2.03 | 8.17E-07 | 3.67E-04 |
| TNFAIP6 | tumor necrosis factor. alpha-induced protein 6 | 4.80 | 1.08E-04 | 3.52E-03 |
| TNFRSF21 | tumor necrosis factor receptor superfamily. member 21 | -1.55 | 1.40E-03 | 1.64E-02 |
| TNFRSF9 | tumor necrosis factor receptor superfamily. member 9 | 1.57 | 8.26E-05 | 3.03E-03 |
| TNIP1 | TNFAIP3 interacting protein 1 | 1.93 | 5.36E-05 | 2.42E-03 |
| TNIP3 | TNFAIP3 interacting protein 3 | 2.56 | 6.03E-06 | 8.73E-04 |
| TRAF1 | TNF receptor-associated factor 1 | 2.25 | 8.97E-07 | 3.78E-04 |
| WTAP | Wilms tumor 1 associated protein | 1.90 | 1.87E-06 | 5.09E-04 |
| ZC3H12A | zinc finger CCCH-type containing 12A | 1.92 | 1.09E-06 | 3.82E-04 |
| ZSWIM4 | zinc finger. SWIM-type containing 4 | 1.56 | 2.63E-04 | 5.90E-03 |

List of DEGs in AMCT infected with Mtb UT205 compared to AMCT-NI

| **Gene** | **Gene Name** | **LogFC** | **P.Value** | **FDR** |
| --- | --- | --- | --- | --- |
| ADM | adrenomedullin | 1.67 | 8.74E-05 | 7.43E-03 |
| APOL3 | apolipoprotein L. 3 | 2.71 | 1.35E-05 | 3.24E-03 |
| BCL3 | B-cell CLL/lymphoma 3 | 1.65 | 1.92E-04 | 1.02E-02 |
| BIRC3 | baculoviral IAP repeat containing 3 | 2.68 | 1.32E-05 | 3.24E-03 |
| BTG1 | B-cell translocation gene 1. anti-proliferative | 2.01 | 4.26E-04 | 1.51E-02 |
| BTG3 | BTG family. member 3 | 1.60 | 1.39E-04 | 8.60E-03 |
| CABLES1 | Cdk5 and Abl enzyme substrate 1 | -1.81 | 2.11E-05 | 3.57E-03 |
| CCL2 | chemokine (C-C motif) ligand 2 | 2.06 | 1.97E-04 | 1.02E-02 |
| CCL20 | chemokine (C-C motif) ligand 20 | 4.60 | 1.13E-03 | 2.63E-02 |
| CCL3 | chemokine (C-C motif) ligand 3 | 4.20 | 4.08E-05 | 4.56E-03 |
| CCL3L1 | chemokine (C-C motif) ligand 3-like 1 | 4.05 | 3.31E-05 | 4.09E-03 |
| CCL3L3 | chemokine (C-C motif) ligand 3-like 3 | 3.84 | 2.24E-04 | 1.10E-02 |
| CCL4L1 | chemokine (C-C motif) ligand 4-like 1 | 5.09 | 2.73E-04 | 1.18E-02 |
| CCL4L2 | chemokine (C-C motif) ligand 4-like 2 | 4.55 | 2.67E-04 | 1.18E-02 |
| CCL5 | chemokine (C-C motif) ligand 5 | 3.50 | 8.63E-05 | 7.42E-03 |
| CCL8 | chemokine (C-C motif) ligand 8 | 5.08 | 8.35E-06 | 2.91E-03 |
| CCR7 | chemokine (C-C motif) receptor 7 | 1.85 | 2.42E-04 | 1.15E-02 |
| CD40 | CD40 molecule. TNF receptor superfamily member 5 | 1.80 | 2.30E-06 | 1.91E-03 |
| CD80 | CD80 molecule | 2.10 | 2.02E-06 | 1.91E-03 |
| CXCL1 | chemokine (C-X-C motif) ligand 1 (melanoma growth stimulating activity. alpha) | 3.22 | 3.64E-05 | 4.29E-03 |
| CXCL10 | chemokine (C-X-C motif) ligand 10 | 5.45 | 1.61E-05 | 3.34E-03 |
| CXCL11 | chemokine (C-X-C motif) ligand 11 | 1.96 | 1.29E-03 | 2.84E-02 |
| CXCL5 | chemokine (C-X-C motif) ligand 5 | 1.64 | 1.32E-03 | 2.86E-02 |
| CXCL8 | chemokine (C-X-C motif) ligand 8 | 4.55 | 2.45E-03 | 3.95E-02 |
| CYP27B1 | cytochrome P450. family 27. subfamily B. polypeptide 1 | 1.86 | 4.34E-04 | 1.52E-02 |
| DUSP5 | dual specificity phosphatase 5 | 1.52 | 2.44E-05 | 3.67E-03 |
| EDN1 | endothelin 1 | 2.40 | 1.89E-06 | 1.91E-03 |
| EHD1 | EH-domain containing 1 | 2.21 | 1.04E-05 | 3.14E-03 |
| FSCN1 | fascin actin-bundling protein 1 | 2.34 | 2.12E-03 | 3.64E-02 |
| G0S2 | G0/G1 switch 2 | 1.72 | 5.97E-04 | 1.87E-02 |
| GBP1 | guanylate binding protein 1. interferon-inducible | 3.42 | 2.61E-05 | 3.86E-03 |
| GBP1P1 | guanylate binding protein 1. interferon-inducible pseudogene 1 | 2.00 | 1.39E-03 | 2.92E-02 |
| GBP2 | guanylate binding protein 2. interferon-inducible | 1.58 | 1.39E-04 | 8.60E-03 |
| GBP4 | guanylate binding protein 4 | 3.00 | 1.36E-04 | 8.56E-03 |
| GBP5 | guanylate binding protein 5 | 3.85 | 1.17E-04 | 8.16E-03 |
| GCH1 | GTP cyclohydrolase 1 | 2.82 | 1.90E-06 | 1.91E-03 |
| GPR34 | G protein-coupled receptor 34 | -1.61 | 2.45E-04 | 1.15E-02 |
| GRAMD1A | GRAM domain containing 1A | 2.29 | 3.94E-05 | 4.49E-03 |
| HCK | HCK proto-oncogene. Src family tyrosine kinase | 1.64 | 1.81E-04 | 9.91E-03 |
| ICAM1 | intercellular adhesion molecule 1 | 2.25 | 4.84E-05 | 5.21E-03 |
| IDO1 | indoleamine 2.3-dioxygenase 1 | 2.24 | 1.68E-03 | 3.23E-02 |
| IER3 | immediate early response 3 | 3.26 | 9.19E-05 | 7.60E-03 |
| IFNGR2 | interferon gamma receptor 2 | 1.56 | 1.24E-05 | 3.24E-03 |
| IL10RA | interleukin 10 receptor. alpha | 1.58 | 1.73E-05 | 3.34E-03 |
| IL15RA | interleukin 15 receptor. alpha | 1.59 | 1.78E-07 | 8.92E-04 |
| IL1A | interleukin 1. alpha | 2.79 | 2.49E-04 | 1.16E-02 |
| IL1B | interleukin 1. beta | 4.14 | 1.42E-03 | 2.94E-02 |
| IL4I1 | interleukin 4 induced 1 | 1.58 | 4.09E-05 | 4.56E-03 |
| IL6 | interleukin 6 | 3.86 | 4.43E-05 | 4.91E-03 |
| IL7R | interleukin 7 receptor | 3.08 | 1.50E-04 | 9.01E-03 |
| IRAK2 | interleukin-1 receptor-associated kinase 2 | 2.24 | 3.46E-05 | 4.17E-03 |
| IRF1 | interferon regulatory factor 1 | 2.47 | 2.54E-04 | 1.17E-02 |
| ISG20 | interferon stimulated exonuclease gene 20kDa | 2.43 | 3.29E-03 | 4.75E-02 |
| ITGB8 | integrin. beta 8 | 1.56 | 1.50E-05 | 3.24E-03 |
| KYNU | kynureninase | 1.51 | 6.77E-04 | 1.99E-02 |
| MARCKS | myristoylated alanine-rich protein kinase C substrate | 2.01 | 1.42E-03 | 2.94E-02 |
| MCOLN2 | mucolipin 2 | 2.61 | 1.77E-05 | 3.34E-03 |
| MYO1G | myosin IG | 1.56 | 1.50E-03 | 3.02E-02 |
| NAMPT | nicotinamide phosphoribosyltransferase | 2.10 | 3.05E-04 | 1.29E-02 |
| NBN | nibrin | 2.17 | 3.95E-06 | 2.38E-03 |
| NEURL3 | neuralized E3 ubiquitin protein ligase 3 | 2.34 | 2.49E-04 | 1.16E-02 |
| NFKB1 | nuclear factor of kappa light polypeptide gene enhancer in B-cells 1 | 2.06 | 1.49E-05 | 3.24E-03 |
| NFKBIA | nuclear factor of kappa light polypeptide gene enhancer in B-cells inhibitor. alpha | 2.16 | 1.04E-04 | 7.85E-03 |
| NFKBIE | nuclear factor of kappa light polypeptide gene enhancer in B-cells inhibitor. epsilon | 1.59 | 5.23E-04 | 1.72E-02 |
| NFKBIZ | nuclear factor of kappa light polypeptide gene enhancer in B-cells inhibitor. zeta | 2.08 | 1.03E-05 | 3.14E-03 |
| P2RX7 | purinergic receptor P2X. ligand-gated ion channel. 7 | 1.92 | 8.04E-05 | 7.07E-03 |
| PDE4B | phosphodiesterase 4B. cAMP-specific | 2.25 | 3.58E-06 | 2.25E-03 |
| PDK4 | pyruvate dehydrogenase kinase. isozyme 4 | -1.82 | 1.74E-04 | 9.74E-03 |
| PSTPIP2 | proline-serine-threonine phosphatase interacting protein 2 | 2.42 | 2.76E-05 | 3.87E-03 |
| PTGS2 | prostaglandin-endoperoxide synthase 2 (prostaglandin G/H synthase and cyclooxygenase) | 3.15 | 2.14E-04 | 1.07E-02 |
| RIPK2 | receptor-interacting serine-threonine kinase 2 | 1.89 | 1.58E-07 | 8.92E-04 |
| RNF144B | ring finger protein 144B | 1.96 | 7.63E-06 | 2.87E-03 |
| RNF19B | ring finger protein 19B | 1.90 | 3.35E-06 | 2.19E-03 |
| SLAMF7 | SLAM family member 7 | 1.56 | 2.38E-05 | 3.67E-03 |
| SLC25A24 | solute carrier family 25 (mitochondrial carrier; phosphate carrier). member 24 | 2.12 | 3.72E-04 | 1.41E-02 |
| SLC2A6 | solute carrier family 2 (facilitated glucose transporter). member 6 | 3.03 | 1.48E-05 | 3.24E-03 |
| SLC39A8 | solute carrier family 39 (zinc transporter). member 8 | 2.26 | 4.81E-04 | 1.62E-02 |
| SOD2 | superoxide dismutase 2. mitochondrial | 3.13 | 8.13E-06 | 2.91E-03 |
| TAP1 | transporter 1. ATP-binding cassette. sub-family B (MDR/TAP) | 2.04 | 3.01E-05 | 3.94E-03 |
| TBC1D2 | TBC1 domain family. member 2 | -1.61 | 4.17E-04 | 1.48E-02 |
| TNF | tumor necrosis factor | 4.16 | 1.39E-05 | 3.24E-03 |
| TNFAIP2 | tumor necrosis factor. alpha-induced protein 2 | 1.53 | 5.72E-04 | 1.83E-02 |
| TNFAIP3 | tumor necrosis factor. alpha-induced protein 3 | 2.08 | 2.78E-06 | 1.96E-03 |
| TNFAIP6 | tumor necrosis factor. alpha-induced protein 6 | 4.65 | 4.85E-04 | 1.62E-02 |
| TNFRSF21 | tumor necrosis factor receptor superfamily. member 21 | -1.52 | 1.67E-03 | 3.22E-02 |
| TNFRSF9 | tumor necrosis factor receptor superfamily. member 9 | 1.53 | 2.33E-05 | 3.67E-03 |
| TNIP1 | TNFAIP3 interacting protein 1 | 1.74 | 1.14E-04 | 8.16E-03 |
| TNIP3 | TNFAIP3 interacting protein 3 | 2.29 | 5.84E-05 | 5.94E-03 |
| TRAF1 | TNF receptor-associated factor 1 | 2.21 | 4.86E-06 | 2.44E-03 |
| WTAP | Wilms tumor 1 associated protein | 1.75 | 4.11E-07 | 1.41E-03 |
| ZC3H12A | zinc finger CCCH-type containing 12A | 2.04 | 6.63E-07 | 1.41E-03 |

List of DEGs in AMTB infected with Mtb UT127 compared to AMCT-NI

| **Gene** | **Gene Name** | **LogFC** | **P.Value** | **FDR** |
| --- | --- | --- | --- | --- |
| ADM | adrenomedullin | 1.60 | 1.16E-03 | 2.84E-02 |
| AIM2 | absent in melanoma 2 | 1.86 | 2.98E-04 | 1.29E-02 |
| ANKRD22 | ankyrin repeat domain 22 | 2.99 | 6.11E-04 | 2.02E-02 |
| APOL3 | apolipoprotein L. 3 | 2.72 | 1.34E-04 | 8.70E-03 |
| ARID5B | AT rich interactive domain 5B (MRF1-like) | 1.51 | 2.23E-03 | 3.98E-02 |
| B4GALT5 | UDP-Gal:betaGlcNAc beta 1.4- galactosyltransferase. polypeptide 5 | 1.56 | 1.07E-04 | 7.57E-03 |
| BCL3 | B-cell CLL/lymphoma 3 | 1.54 | 1.38E-05 | 2.68E-03 |
| BIRC3 | baculoviral IAP repeat containing 3 | 2.86 | 6.20E-05 | 6.02E-03 |
| BTG1 | B-cell translocation gene 1. anti-proliferative | 2.30 | 2.94E-04 | 1.29E-02 |
| CCL20 | chemokine (C-C motif) ligand 20 | 5.29 | 4.58E-07 | 7.48E-04 |
| CCL3 | chemokine (C-C motif) ligand 3 | 4.45 | 2.79E-06 | 1.51E-03 |
| CCL3L1 | chemokine (C-C motif) ligand 3-like 1 | 4.37 | 2.83E-06 | 1.51E-03 |
| CCL3L3 | chemokine (C-C motif) ligand 3-like 3 | 4.06 | 2.63E-06 | 1.51E-03 |
| CCL4L1 | chemokine (C-C motif) ligand 4-like 1 | 5.24 | 2.10E-06 | 1.51E-03 |
| CCL4L2 | chemokine (C-C motif) ligand 4-like 2 | 3.75 | 1.45E-04 | 9.14E-03 |
| CCL5 | chemokine (C-C motif) ligand 5 | 3.71 | 2.96E-04 | 1.29E-02 |
| CCL8 | chemokine (C-C motif) ligand 8 | 4.64 | 3.87E-04 | 1.54E-02 |
| CCR7 | chemokine (C-C motif) receptor 7 | 3.35 | 1.13E-05 | 2.53E-03 |
| CD274 | CD274 molecule | 1.93 | 4.87E-07 | 7.48E-04 |
| CD40 | CD40 molecule. TNF receptor superfamily member 5 | 2.17 | 2.18E-04 | 1.10E-02 |
| CD80 | CD80 molecule | 1.93 | 1.26E-05 | 2.60E-03 |
| CD83 | CD83 molecule | 1.58 | 5.40E-05 | 5.75E-03 |
| CKB | creatine kinase. brain | 1.98 | 2.58E-05 | 3.76E-03 |
| CSRNP1 | cysteine-serine-rich nuclear protein 1 | 1.74 | 4.22E-06 | 1.63E-03 |
| CXCL10 | chemokine (C-X-C motif) ligand 10 | 6.04 | 2.50E-08 | 1.26E-04 |
| CXCL11 | chemokine (C-X-C motif) ligand 11 | 2.75 | 4.25E-06 | 1.63E-03 |
| CXCL8 | chemokine (C-X-C motif) ligand 8 | 4.78 | 1.03E-05 | 2.53E-03 |
| CXCL9 | chemokine (C-X-C motif) ligand 9 | 4.82 | 1.60E-06 | 1.51E-03 |
| DENND5A | DENN/MADD domain containing 5A | 1.51 | 3.60E-05 | 4.36E-03 |
| DUSP5 | dual specificity phosphatase 5 | 1.53 | 9.94E-04 | 2.60E-02 |
| EBI3 | Epstein-Barr virus induced 3 | 1.64 | 3.32E-04 | 1.40E-02 |
| EHD1 | EH-domain containing 1 | 1.82 | 1.12E-05 | 2.53E-03 |
| EPSTI1 | epithelial stromal interaction 1 (breast) | 2.07 | 1.30E-05 | 2.60E-03 |
| FCGR1A | Fc fragment of IgG. high affinity Ia. receptor (CD64) | 1.54 | 1.10E-04 | 7.69E-03 |
| GBP1 | guanylate binding protein 1. interferon-inducible | 4.07 | 2.63E-07 | 6.65E-04 |
| GBP1P1 | guanylate binding protein 1. interferon-inducible pseudogene 1 | 2.37 | 1.15E-08 | 8.72E-05 |
| GBP4 | guanylate binding protein 4 | 3.77 | 9.93E-05 | 7.19E-03 |
| GBP5 | guanylate binding protein 5 | 4.84 | 2.31E-05 | 3.67E-03 |
| GCH1 | GTP cyclohydrolase 1 | 3.07 | 7.23E-06 | 2.23E-03 |
| GK | glycerol kinase | 1.54 | 1.10E-03 | 2.75E-02 |
| GPR132 | G protein-coupled receptor 132 | 1.83 | 6.46E-04 | 2.10E-02 |
| GPR34 | G protein-coupled receptor 34 | -1.57 | 8.26E-04 | 2.36E-02 |
| GRAMD1A | GRAM domain containing 1A | 2.04 | 3.50E-05 | 4.32E-03 |
| HELZ2 | helicase with zinc finger 2. transcriptional coactivator | 1.99 | 6.24E-06 | 2.05E-03 |
| ICAM1 | intercellular adhesion molecule 1 | 2.81 | 3.78E-06 | 1.63E-03 |
| IDO1 | indoleamine 2.3-dioxygenase 1 | 4.38 | 1.67E-04 | 9.96E-03 |
| IER3 | immediate early response 3 | 3.73 | 2.94E-06 | 1.51E-03 |
| IFI44L | interferon-induced protein 44-like | 1.55 | 1.15E-04 | 7.90E-03 |
| IFIH1 | interferon induced with helicase C domain 1 | 1.55 | 1.10E-05 | 2.53E-03 |
| IFIT2 | interferon-induced protein with tetratricopeptide repeats 2 | 2.22 | 5.11E-04 | 1.84E-02 |
| IFIT3 | interferon-induced protein with tetratricopeptide repeats 3 | 2.35 | 2.52E-05 | 3.76E-03 |
| IL10RA | interleukin 10 receptor. alpha | 1.76 | 1.65E-05 | 3.01E-03 |
| IL15 | interleukin 15 | 1.73 | 9.68E-05 | 7.14E-03 |
| IL15RA | interleukin 15 receptor. alpha | 1.73 | 2.85E-06 | 1.51E-03 |
| IL1A | interleukin 1. alpha | 2.86 | 3.09E-04 | 1.33E-02 |
| IL1B | interleukin 1. beta | 5.52 | 1.33E-06 | 1.42E-03 |
| IL23A | interleukin 23. alpha subunit p19 | 2.95 | 2.08E-04 | 1.08E-02 |
| IL27 | interleukin 27 | 1.89 | 6.00E-05 | 5.94E-03 |
| IL6 | interleukin 6 | 3.82 | 4.65E-04 | 1.72E-02 |
| IL7R | interleukin 7 receptor | 2.59 | 2.43E-04 | 1.18E-02 |
| IRAK2 | interleukin-1 receptor-associated kinase 2 | 1.79 | 2.61E-05 | 3.76E-03 |
| IRF1 | interferon regulatory factor 1 | 2.69 | 1.31E-05 | 2.60E-03 |
| ISG20 | interferon stimulated exonuclease gene 20kDa | 2.94 | 2.76E-05 | 3.84E-03 |
| LAMP3 | lysosomal-associated membrane protein 3 | 2.23 | 7.36E-05 | 6.53E-03 |
| MCOLN2 | mucolipin 2 | 2.36 | 2.66E-03 | 4.47E-02 |
| MTHFD2 | methylenetetrahydrofolate dehydrogenase (NADP+ dependent) 2. methenyltetrahydrofolate cyclohydrolase | 1.63 | 4.99E-06 | 1.80E-03 |
| MX1 | MX dynamin-like GTPase 1 | 1.74 | 3.08E-05 | 4.05E-03 |
| NAMPT | nicotinamide phosphoribosyltransferase | 2.13 | 2.89E-05 | 3.89E-03 |
| NBN | nibrin | 1.77 | 4.24E-05 | 4.78E-03 |
| NEURL3 | neuralized E3 ubiquitin protein ligase 3 | 2.57 | 1.72E-05 | 3.09E-03 |
| NFKB1 | nuclear factor of kappa light polypeptide gene enhancer in B-cells 1 | 1.83 | 4.19E-06 | 1.63E-03 |
| NFKBIA | nuclear factor of kappa light polypeptide gene enhancer in B-cells inhibitor. alpha | 1.99 | 1.49E-04 | 9.34E-03 |
| NFKBIZ | nuclear factor of kappa light polypeptide gene enhancer in B-cells inhibitor. zeta | 2.49 | 3.77E-04 | 1.51E-02 |
| P2RX7 | purinergic receptor P2X. ligand-gated ion channel. 7 | 1.91 | 2.12E-07 | 6.65E-04 |
| PDE4B | phosphodiesterase 4B. cAMP-specific | 2.34 | 9.86E-06 | 2.53E-03 |
| PDK4 | pyruvate dehydrogenase kinase. isozyme 4 | -1.99 | 1.02E-03 | 2.64E-02 |
| PIM1 | Pim-1 proto-oncogene. serine/threonine kinase | 2.01 | 2.16E-04 | 1.09E-02 |
| PSTPIP2 | proline-serine-threonine phosphatase interacting protein 2 | 2.54 | 2.10E-05 | 3.49E-03 |
| PTGS2 | prostaglandin-endoperoxide synthase 2 (prostaglandin G/H synthase and cyclooxygenase) | 3.74 | 4.43E-04 | 1.68E-02 |
| RFTN1 | raftlin. lipid raft linker 1 | 1.65 | 9.39E-04 | 2.53E-02 |
| RIPK2 | receptor-interacting serine-threonine kinase 2 | 1.93 | 6.13E-06 | 2.05E-03 |
| RNF144B | ring finger protein 144B | 1.67 | 5.82E-05 | 5.94E-03 |
| RNF19B | ring finger protein 19B | 2.12 | 2.46E-07 | 6.65E-04 |
| RSAD2 | radical S-adenosyl methionine domain containing 2 | 3.03 | 8.37E-06 | 2.39E-03 |
| SLAMF1 | signaling lymphocytic activation molecule family member 1 | 3.25 | 9.83E-05 | 7.15E-03 |
| SLAMF7 | SLAM family member 7 | 1.90 | 9.59E-04 | 2.56E-02 |
| SLC25A24 | solute carrier family 25 (mitochondrial carrier; phosphate carrier). member 24 | 1.80 | 1.95E-04 | 1.05E-02 |
| SLC2A6 | solute carrier family 2 (facilitated glucose transporter). member 6 | 3.03 | 2.38E-05 | 3.70E-03 |
| SOCS1 | suppressor of cytokine signaling 1 | 1.68 | 2.45E-06 | 1.51E-03 |
| SOD2 | superoxide dismutase 2. mitochondrial | 2.90 | 3.20E-05 | 4.13E-03 |
| STAT4 | signal transducer and activator of transcription 4 | 1.54 | 8.83E-04 | 2.44E-02 |
| STX11 | syntaxin 11 | 1.57 | 2.81E-05 | 3.84E-03 |
| TAP1 | transporter 1. ATP-binding cassette. sub-family B (MDR/TAP) | 2.00 | 4.79E-04 | 1.76E-02 |
| TMEM194A | transmembrane protein 194A | 1.57 | 4.87E-05 | 5.27E-03 |
| TNF | tumor necrosis factor | 5.10 | 6.91E-06 | 2.18E-03 |
| TNFAIP2 | tumor necrosis factor. alpha-induced protein 2 | 1.80 | 4.21E-05 | 4.78E-03 |
| TNFAIP3 | tumor necrosis factor. alpha-induced protein 3 | 1.75 | 1.63E-04 | 9.88E-03 |
| TNFAIP6 | tumor necrosis factor. alpha-induced protein 6 | 4.55 | 8.90E-09 | 8.72E-05 |
| TNFAIP8 | tumor necrosis factor. alpha-induced protein 8 | 1.69 | 2.35E-04 | 1.16E-02 |
| TNFRSF9 | tumor necrosis factor receptor superfamily. member 9 | 1.69 | 1.21E-05 | 2.58E-03 |
| TNFSF10 | tumor necrosis factor (ligand) superfamily. member 10 | 1.55 | 1.35E-03 | 3.07E-02 |
| TNIP1 | TNFAIP3 interacting protein 1 | 1.75 | 2.08E-04 | 1.08E-02 |
| TRAF1 | TNF receptor-associated factor 1 | 2.25 | 1.92E-04 | 1.05E-02 |
| WARS | tryptophanyl-tRNA synthetase | 2.31 | 1.43E-03 | 3.14E-02 |
| ZC3H12A | zinc finger CCCH-type containing 12A | 2.21 | 3.64E-05 | 4.37E-03 |

List of DEGs in AMTB infected with Mtb UT205 compared to AMCT-NI

| **Gene** | **Gene Name** | **LogFC** | **P.Value** | **FDR** |
| --- | --- | --- | --- | --- |
| APOL3 | apolipoprotein L. 3 | 2.19 | 4.42E-03 | 1.53E-02 |
| BIRC3 | baculoviral IAP repeat containing 3 | 1.98 | 5.96E-04 | 6.65E-03 |
| CCL2 | chemokine (C-C motif) ligand 2 | 1.83 | 4.82E-02 | 3.92E-02 |
| CCL20 | chemokine (C-C motif) ligand 20 | 4.00 | 3.78E-09 | 5.47E-05 |
| CCL3 | chemokine (C-C motif) ligand 3 | 3.46 | 1.51E-04 | 4.61E-02 |
| CCL3L3 | chemokine (C-C motif) ligand 3-like 3 | 3.44 | 9.55E-05 | 3.66E-02 |
| CCL4L1 | chemokine (C-C motif) ligand 4-like 1 | 4.39 | 3.97E-05 | 2.73E-02 |
| CCL4L2 | chemokine (C-C motif) ligand 4-like 2 | 3.70 | 3.51E-04 | 5.37E-03 |
| CCL5 | chemokine (C-C motif) ligand 5 | 2.57 | 3.29E-03 | 1.39E-02 |
| CCL8 | chemokine (C-C motif) ligand 8 | 4.57 | 6.07E-04 | 6.65E-03 |
| CCR7 | chemokine (C-C motif) receptor 7 | 1.70 | 2.91E-03 | 1.33E-02 |
| CSF2 | colony stimulating factor 2 (granulocyte-macrophage) | 1.64 | 5.94E-02 | 4.22E-02 |
| CSRNP1 | cysteine-serine-rich nuclear protein 1 | 1.68 | 1.72E-05 | 2.08E-02 |
| CXCL10 | chemokine (C-X-C motif) ligand 10 | 5.70 | 1.75E-06 | 8.43E-03 |
| CXCL11 | chemokine (C-X-C motif) ligand 11 | 1.81 | 4.90E-02 | 3.94E-02 |
| CXCL8 | chemokine (C-X-C motif) ligand 8 | 4.04 | 1.44E-04 | 4.61E-02 |
| CXCL9 | chemokine (C-X-C motif) ligand 9 | 3.14 | 2.55E-03 | 1.28E-02 |
| EPSTI1 | epithelial stromal interaction 1 (breast) | 1.74 | 1.32E-03 | 9.52E-03 |
| GBP1 | guanylate binding protein 1. interferon-inducible | 3.29 | 2.81E-04 | 5.31E-03 |
| GBP1P1 | guanylate binding protein 1. interferon-inducible pseudogene 1 | 1.71 | 7.17E-04 | 7.12E-03 |
| GBP4 | guanylate binding protein 4 | 2.77 | 1.20E-02 | 2.36E-02 |
| GBP5 | guanylate binding protein 5 | 3.69 | 3.54E-03 | 1.42E-02 |
| GCH1 | GTP cyclohydrolase 1 | 2.36 | 3.33E-04 | 5.31E-03 |
| HELZ2 | helicase with zinc finger 2. transcriptional coactivator | 1.86 | 9.33E-06 | 1.68E-02 |
| ICAM1 | intercellular adhesion molecule 1 | 1.85 | 3.23E-04 | 5.31E-03 |
| IDO1 | indoleamine 2.3-dioxygenase 1 | 2.32 | 3.34E-02 | 3.41E-02 |
| IER3 | immediate early response 3 | 2.35 | 1.39E-03 | 9.75E-03 |
| IFIT1 | interferon-induced protein with tetratricopeptide repeats 1 | 1.93 | 5.65E-02 | 4.18E-02 |
| IFIT2 | interferon-induced protein with tetratricopeptide repeats 2 | 2.90 | 6.08E-04 | 6.65E-03 |
| IFIT3 | interferon-induced protein with tetratricopeptide repeats 3 | 2.40 | 8.49E-05 | 3.61E-02 |
| IFNG | interferon. gamma | 2.78 | 4.30E-02 | 3.78E-02 |
| IL1A | interleukin 1. alpha | 1.57 | 3.21E-03 | 1.38E-02 |
| IL1B | interleukin 1. beta | 4.30 | 5.13E-05 | 3.19E-02 |
| IL6 | interleukin 6 | 2.17 | 3.76E-05 | 2.72E-02 |
| IL7R | interleukin 7 receptor | 2.01 | 1.99E-03 | 1.13E-02 |
| IRF1 | interferon regulatory factor 1 | 2.48 | 7.94E-05 | 3.48E-02 |
| ISG15 | ISG15 ubiquitin-like modifier | 1.54 | 7.38E-03 | 1.94E-02 |
| ISG20 | interferon stimulated exonuclease gene 20kDa | 2.50 | 4.92E-04 | 6.23E-02 |
| MARCKS | myristoylated alanine-rich protein kinase C substrate | 1.93 | 2.76E-02 | 3.18E-02 |
| MCOLN2 | mucolipin 2 | 1.56 | 4.65E-03 | 1.57E-02 |
| MX1 | MX dynamin-like GTPase 1 | 1.74 | 6.17E-05 | 3.30E-02 |
| NAMPT | nicotinamide phosphoribosyltransferase | 1.75 | 9.63E-05 | 3.66E-02 |
| NEURL3 | neuralized E3 ubiquitin protein ligase 3 | 2.10 | 2.21E-04 | 4.93E-02 |
| NFKBIA | nuclear factor of kappa light polypeptide gene enhancer in B-cells inhibitor. alpha | 1.67 | 5.34E-04 | 6.44E-03 |
| NFKBIZ | nuclear factor of kappa light polypeptide gene enhancer in B-cells inhibitor. zeta | 1.62 | 1.96E-04 | 4.93E-02 |
| P2RX7 | purinergic receptor P2X. ligand-gated ion channel. 7 | 1.53 | 4.76E-04 | 6.19E-03 |
| PDK4 | pyruvate dehydrogenase kinase. isozyme 4 | -1.58 | 5.62E-03 | 1.70E-02 |
| PSTPIP2 | proline-serine-threonine phosphatase interacting protein 2 | 1.94 | 2.58E-03 | 1.28E-02 |
| PTGS2 | prostaglandin-endoperoxide synthase 2 (prostaglandin G/H synthase and cyclooxygenase) | 1.84 | 6.00E-04 | 6.65E-03 |
| RNF144B | ring finger protein 144B | 1.53 | 3.33E-04 | 5.31E-03 |
| RNF19B | ring finger protein 19B | 1.65 | 3.47E-05 | 2.64E-02 |
| RSAD2 | radical S-adenosyl methionine domain containing 2 | 2.75 | 6.65E-05 | 3.41E-02 |
| SERPINE2 | serpin peptidase inhibitor. clade E (nexin. plasminogen activator inhibitor type 1). member 2 | 1.95 | 3.93E-02 | 3.65E-02 |
| SLAMF1 | signaling lymphocytic activation molecule family member 1 | 1.52 | 2.30E-02 | 2.95E-02 |
| SLC2A6 | solute carrier family 2 (facilitated glucose transporter). member 6 | 2.46 | 2.87E-04 | 5.31E-03 |
| SOCS1 | suppressor of cytokine signaling 1 | 1.54 | 2.82E-06 | 9.31E-03 |
| SOD2 | superoxide dismutase 2. mitochondrial | 2.25 | 2.20E-04 | 4.93E-02 |
| TAP1 | transporter 1. ATP-binding cassette. sub-family B (MDR/TAP) | 1.56 | 1.15E-02 | 2.32E-02 |
| TNF | tumor necrosis factor | 4.05 | 3.31E-05 | 2.64E-02 |
| TNFAIP6 | tumor necrosis factor. alpha-induced protein 6 | 3.87 | 8.86E-06 | 1.68E-02 |

List of DEGs in SMs infected with Mtb UT127 compared to SMs-NI

| **Gene** | **Gene Name** | **LogFC** | **P.Value** | **FDR** |
| --- | --- | --- | --- | --- |
| BIRC3 | baculoviral IAP repeat containing 3 | 1.68 | 1.56E-02 | 3.79E-01 |
| CCL3 | chemokine (C-C motif) ligand 3 | 2.17 | 6.31E-03 | 4.74E-01 |
| CCL3L1 | chemokine (C-C motif) ligand 3-like 1 | 2.18 | 4.45E-02 | 4.37E-01 |
| CCL3L3 | chemokine (C-C motif) ligand 3-like 3 | 1.93 | 6.81E-03 | 4.80E-01 |
| CCL4L1 | chemokine (C-C motif) ligand 4-like 1 | 2.35 | 7.00E-02 | 4.85E-01 |
| CCL4L2 | chemokine (C-C motif) ligand 4-like 2 | 2.15 | 2.65E-02 | 4.05E-01 |
| CCL5 | chemokine (C-C motif) ligand 5 | 1.89 | 1.14E-03 | 3.26E-01 |
| CCR7 | chemokine (C-C motif) receptor 7 | 1.55 | 9.43E-03 | 3.49E-01 |
| CXCL8 | chemokine (C-X-C motif) ligand 8 | 2.09 | 1.32E-02 | 5.59E-01 |
| IER3 | immediate early response 3 | 1.51 | 1.98E-02 | 6.12E-01 |
| IL1B | interleukin 1. beta | 2.88 | 1.35E-02 | 5.61E-01 |
| MARCKS | myristoylated alanine-rich protein kinase C substrate | 1.63 | 4.79E-02 | 4.41E-01 |
| MCOLN2 | mucolipin 2 | 1.51 | 1.59E-02 | 3.80E-01 |
| PTGS2 | prostaglandin-endoperoxide synthase 2 | 1.96 | 1.25E-02 | 5.51E-01 |
| SERPINB2 | serpin peptidase inhibitor. clade B (ovalbumin). member 2 | 2.05 | 1.45E-02 | 5.69E-01 |
| SLC2A6 | solute carrier family 2 (facilitated glucose transporter). member 6 | 1.66 | 1.75E-02 | 3.80E-01 |
| TNF | tumor necrosis factor | 1.96 | 1.32E-02 | 5.59E-01 |
| TNFAIP6 | tumor necrosis factor. alpha-induced protein 6 | 2.19 | 1.14E-02 | 5.41E-01 |
