# Supplementary material for: Human Alveolar and Splenic Macrophage Populations Display a Distinct Transcriptomic Response to Infection With Mycobacterium tuberculosis
Source: Front Immunol. 2020 Apr 21;11:630. doi: 10.3389/fimmu.2020.00630 (PMC7186480; doi:10.3389/fimmu.2020.00630)
Supplement: Supplementary file 3 [file Table_2.DOCX]

**Supplementary table 2:** Analysis comparing the list of DEGs expressed between AMCT and AMTB infected with the clinical isolates of Mtb UT127 and UT205. Up-regulated genes and down-regulated genes are highlighted in red and green respectively.

| **Genes** | **Log2**  **AMCT-127** | **Log2 AMCT-205** | **Log2 AMTB-127** | **Log2 AMTB-205** |
| --- | --- | --- | --- | --- |
| **Core transcriptome Alveolar macrophages-Mtb**  **(67 genes)** | | | | |
| **APOL3** | 2.7 | 2.1 | 2.7 | 2.2 |
| **BIRC3** | 2.7 | 2.5 | 2.9 | 2.0 |
| **CCL20** | 4.6 | 5.1 | 5.3 | 4.0 |
| **CCL3** | 4.2 | 4.1 | 4.4 | 3.5 |
| **CCL3L3** | 3.8 | 3.8 | 4.1 | 3.4 |
| **CCL4L1** | 5.1 | 5.1 | 5.2 | 4.4 |
| **CCL4L2** | 4.5 | 4.6 | 3.8 | 3.7 |
| **CCL5** | 3.5 | 3.7 | 3.7 | 2.6 |
| **CCL8** | 5.1 | 4.1 | 4.6 | 4.6 |
| **CCR7** | 1.9 | 2.2 | 3.4 | 1.7 |
| **CXCL10** | 5.5 | 4.8 | 6.0 | 5.7 |
| **CXCL8** | 4.5 | 4.7 | 4.8 | 4.0 |
| **GBP1** | 3.4 | 3.4 | 4.1 | 3.3 |
| **GBP1P1** | 2.0 | 1.8 | 2.4 | 1.7 |
| **GBP4** | 3.0 | 2.6 | 3.8 | 2.8 |
| **GBP5** | 3.9 | 3.6 | 4.8 | 3.7 |
| **GCH1** | 2.8 | 2.8 | 3.1 | 2.4 |
| **ICAM1** | 2.3 | 2.1 | 2.8 | 1.8 |
| **IDO1** | 2.2 | 2.0 | 4.4 | 2.3 |
| **IER3** | 3.3 | 3.3 | 3.7 | 2.3 |
| **IL1A** | 2.8 | 3.1 | 2.9 | 1.6 |
| **IL1B** | 4.1 | 4.4 | 5.5 | 4.3 |
| **IL6** | 3.9 | 4.0 | 3.8 | 2.2 |
| **IL7R** | 3.1 | 3.3 | 2.6 | 2.0 |
| **IRF1** | 2.5 | 2.3 | 2.7 | 2.5 |
| **ISG20** | 2.4 | 1.6 | 2.9 | 2.5 |
| **MCOLN2** | 2.6 | 2.6 | 2.4 | 1.6 |
| **NAMPT** | 2.1 | 2.2 | 2.1 | 1.8 |
| **NEURL3** | 2.3 | 2.0 | 2.6 | 2.1 |
| **NFKBIA** | 2.2 | 2.0 | 2.0 | 1.7 |
| **NFKBIZ** | 2.1 | 2.1 | 2.5 | 1.6 |
| **P2RX7** | 1.9 | 2.0 | 1.9 | 1.5 |
| **PDK4** | -1.8 | -1.6 | -2.0 | -1.6 |
| **PSTPIP2** | 2.4 | 2.6 | 2.5 | 1.9 |
| **PTGS2** | 3.1 | 3.8 | 3.7 | 1.8 |
| **RNF144B** | 2.0 | 1.9 | 1.7 | 1.5 |
| **RNF19B** | 1.9 | 1.6 | 2.1 | 1.7 |
| **SLC2A6** | 3.0 | 3.2 | 3.0 | 2.5 |
| **SOD2** | 3.1 | 3.2 | 2.9 | 2.2 |
| **TAP1** | 2.0 | 1.9 | 2.0 | 1.6 |
| **TNF** | 4.2 | 4.0 | 5.1 | 4.0 |
| **TNFAIP6** | 4.6 | 4.8 | 4.6 | 3.9 |
| **BCL3** | 1.6 | 1.7 | 1.5 |  |
| **BTG1** | 2.0 | 1.8 | 2.3 |  |
| **CCL3L1** | 4.1 | 4.1 | 4.4 |  |
| **CD40** | 1.8 | 1.8 | 2.2 |  |
| **CD80** | 2.1 | 2.2 | 1.9 |  |
| **DUSP5** | 1.5 | 1.6 | 1.5 |  |
| **EHD1** | 2.2 | 2.5 | 1.8 |  |
| **GPR34** | -1.6 | -1.7 | -1.6 |  |
| **GRAMD1A** | 2.3 | 2.4 | 2.0 |  |
| **IRAK2** | 2.2 | 2.4 | 1.8 |  |
| **NBN** | 2.2 | 2.2 | 1.8 |  |
| **NFKB1** | 2.1 | 2.2 | 1.8 |  |
| **PDE4B** | 2.3 | 2.1 | 2.3 |  |
| **RIPK2** | 1.9 | 1.9 | 1.9 |  |
| **SLAMF7** | 1.6 | 1.7 | 1.9 |  |
| **SLC25A24** | 2.1 | 2.3 | 1.8 |  |
| **TNFAIP3** | 2.1 | 2.0 | 1.8 |  |
| **TNFRSF9** | 1.5 | 1.6 | 1.7 |  |
| **TNIP1** | 1.7 | 1.9 | 1.8 |  |
| **TRAF1** | 2.2 | 2.3 | 2.3 |  |
| **ZC3H12A** | 2.0 | 1.9 | 2.2 |  |
| **MARCKS** | 2.0 | 2.1 |  | 1.9 |
| **RSAD2** | 3.0 | 1.6 |  | 2.8 |
| **SLAMF1** | 3.2 | 2.1 |  | 1.5 |
| **CXCL11** | 2.0 |  | 2.7 | 1.8 |
| **Common genes in AMCT-127 and AMCT-205**  **(19 genes)** | | | | |
| **BTG3** | 1.8 | 1.6 |  |  |
| **CABLES1** | -2.0 | -1.8 |  |  |
| **CXCL1** | 3.2 | 3.2 |  |  |
| **CXCL5** | 2.1 | 1.6 |  |  |
| **CYP27B1** | 2.1 | 1.9 |  |  |
| **EDN1** | 2.3 | 2.4 |  |  |
| **FSCN1** | 2.4 | 2.3 |  |  |
| **GBP2** | 1.7 | 1.6 |  |  |
| **HCK** | 1.6 | 1.6 |  |  |
| **IFNGR2** | 1.7 | 1.6 |  |  |
| **ITGB8** | 1.8 | 1.6 |  |  |
| **KYNU** | 1.6 | 1.5 |  |  |
| **MYO1G** | 1.6 | 1.6 |  |  |
| **NFKBIE** | 1.6 | 1.6 |  |  |
| **SLC39A8** | 2.0 | 2.3 |  |  |
| **TBC1D2** | -1.7 | -1.6 |  |  |
| **TNFRSF21** | -1.5 | -1.5 |  |  |
| **TNIP3** | 2.6 | 2.3 |  |  |
| **WTAP** | 1.9 | 1.7 |  |  |
| **Common genes in AMCT-127 and AMTB-127**  **(5 genes)** | | | | |
| **CKB** | 1.8 |  | 2.0 |  |
| **DENND5A** | 1.6 |  | 1.5 |  |
| **EBI3** | 1.6 |  | 1.6 |  |
| **IL23A** | 1.9 |  | 2.9 |  |
| **TMEM194A** | 1.6 |  | 1.6 |  |
| **Common genes in AMCT-205 and AMTB-127**  **(4 genes)** | | | | |
| **ADM** |  | 1.7 | 1.6 |  |
| **IL10RA** |  | 1.6 | 1.8 |  |
| **IL15RA** |  | 1.6 | 1.7 |  |
| **TNFAIP2** |  | 1.5 | 1.8 |  |
| **Common gene in AMCT-205 Vs AMTB-205**  **(1 gene)** | | | | |
| **CCL2** |  | 2.1 |  | 1.8 |
| **Common genes in AMTB-127 and AMTB-205**  **(8 genes)** | | | | |
| **CSRNP1** |  |  | 1.7 | 1.7 |
| **CXCL9** |  |  | 4.8 | 3.1 |
| **EPSTI1** |  |  | 2.1 | 1.7 |
| **HELZ2** |  |  | 2.0 | 1.9 |
| **IFIT2** |  |  | 2.2 | 2.9 |
| **IFIT3** |  |  | 2.3 | 2.4 |
| **MX1** |  |  | 1.7 | 1.7 |
| **SOCS1** |  |  | 1.7 | 1.5 |
| **Unique genes in AMCT-127 (7 genes)** | | | | |
| **ADORA2A** | 1.6 |  |  |  |
| **DHRS3** | -1.5 |  |  |  |
| **FAM129A** | 1.7 |  |  |  |
| **GJB2** | 2.0 |  |  |  |
| **HES2** | -1.7 |  |  |  |
| **PTGER4** | 1.5 |  |  |  |
| **ZSWIM4** | 1.6 |  |  |  |
| **Unique genes in AMCT-205 (2 genes)** | | | | |
| **G0S2** |  | 1.7 |  |  |
| **IL4I1** |  | 1.6 |  |  |
| **Unique genes in AMTB-127 (22 genes)** | | | | |
| **AIM2** |  |  | 1.9 |  |
| **ANKRD22** |  |  | 3.0 |  |
| **ARID5B** |  |  | 1.5 |  |
| **B4GALT5** |  |  | 1.6 |  |
| **CD274** |  |  | 1.9 |  |
| **CD83** |  |  | 1.6 |  |
| **FCGR1A** |  |  | 1.5 |  |
| **GK** |  |  | 1.5 |  |
| **GPR132** |  |  | 1.8 |  |
| **IFI44L** |  |  | 1.5 |  |
| **IFIH1** |  |  | 1.6 |  |
| **IL15** |  |  | 1.7 |  |
| **IL27** |  |  | 1.9 |  |
| **LAMP3** |  |  | 2.2 |  |
| **MTHFD2** |  |  | 1.6 |  |
| **PIM1** |  |  | 2.0 |  |
| **RFTN1** |  |  | 1.6 |  |
| **STAT4** |  |  | 1.5 |  |
| **STX11** |  |  | 1.6 |  |
| **TNFAIP8** |  |  | 1.7 |  |
| **TNFSF10** |  |  | 1.6 |  |
| **WARS** |  |  | 2.3 |  |
| **Unique genes in AMTB-205 (5 genes)** | | | | |
| **CSF2** |  |  |  | 1.6 |
| **IFIT1** |  |  |  | 1.9 |
| **IFNG** |  |  |  | 2.8 |
| **ISG15** |  |  |  | 1.5 |
| **SERPINE2** |  |  |  | 1.9 |
